# Reference genomes of channel catfish and blue catfish reveal multiple pericentric chromosome inversions

**DOI:** 10.1186/s12915-023-01556-8

**Published:** 2023-04-03

**Authors:** Geoffrey C. Waldbieser, Shikai Liu, Zihao Yuan, Caitlin E. Older, Dongya Gao, Chenyu Shi, Brian G. Bosworth, Ning Li, Lisui Bao, Mona A. Kirby, Yulin Jin, Monica L. Wood, Brian Scheffler, Sheron Simpson, Ramey C. Youngblood, Mary V. Duke, Linda Ballard, Adam Phillippy, Sergey Koren, Zhanjiang Liu

**Affiliations:** 1 USDA-ARS Warmwater Aquaculture Research Unit, 141 Experiment Station Road, P.O. Box 38, Stoneville, MS 38776 USA; 2 MOE Key Laboratory of Mariculture and College of Fisheries, Ocean University of China, Qingdao, 266003 China; 3 The Fish Molecular Genetics and Biotechnology Laboratory, School of Fisheries, Aquaculture, and Aquatic Sciences and Program of Cell and Molecular Biosciences, Auburn University, Auburn, AL 36849 USA; 4 Department of Biology, College of Arts and Sciences, Syracuse University, Syracuse, NY 13244 USA; 5 US Department of Agriculture, Agricultural Research Service, Genomics and Bioinformatics Research Unit, Stoneville, MS USA; 6 Institute for Genomics, Biocomputing, and Biotechnology, Mississippi State University, Starkville, MS 39762 USA; 7 Genome Informatics Section, Computational and Statistical Genomics Branch, National Human Genome Research Institute, National Institutes of Health, Bethesda, MD USA

**Keywords:** Genome, Reference genome, Chromosome inversion, Fish, Teleost, Blue catfish, Channel catfish, Aquaculture, Sequence assembly, Speciation

## Abstract

**Background:**

Channel catfish and blue catfish are the most important aquacultured species in the USA. The species do not readily intermate naturally but F_1_ hybrids can be produced through artificial spawning. F_1_ hybrids produced by mating channel catfish female with blue catfish male exhibit heterosis and provide an ideal system to study reproductive isolation and hybrid vigor. The purpose of the study was to generate high-quality chromosome level reference genome sequences and to determine their genomic similarities and differences.

**Results:**

We present high-quality reference genome sequences for both channel catfish and blue catfish, containing only 67 and 139 total gaps, respectively. We also report three pericentric chromosome inversions between the two genomes, as evidenced by long reads across the inversion junctions from distinct individuals, genetic linkage mapping, and PCR amplicons across the inversion junctions. Recombination rates within the inversional segments, detected as double crossovers, are extremely low among backcross progenies (progenies of channel catfish female × F_1_ hybrid male), suggesting that the pericentric inversions interrupt postzygotic recombination or survival of recombinants. Identification of channel catfish- and blue catfish-specific genes, along with expansions of immunoglobulin genes and centromeric *Xba* elements, provides insights into genomic hallmarks of these species.

**Conclusions:**

We generated high-quality reference genome sequences for both blue catfish and channel catfish and identified major chromosomal inversions on chromosomes 6, 11, and 24. These perimetric inversions were validated by additional sequencing analysis, genetic linkage mapping, and PCR analysis across the inversion junctions. The reference genome sequences, as well as the contrasted chromosomal architecture should provide guidance for the interspecific breeding programs.

**Supplementary Information:**

The online version contains supplementary material available at 10.1186/s12915-023-01556-8.

## Background

Catfish belong to the order Siluriformes, the second most diverse vertebrate order containing 39 families with more than 4100 species [1, 2]. Their basal phylogenetic position among teleosts makes them valuable models for comparative biological studies; they are economically important for sport fishing and are third in global aquaculture production, following only the carps and tilapias [3]. Channel catfish (*Ictalurus punctatus*) and blue catfish (*I. furcatus*), native to North America, lead aquaculture production in the USA. The species exhibit differential production and performance traits: channel catfish grow faster in culture but provide lower processing yields than blue catfish; channel catfish is resistant against columnaris disease but susceptible to enteric septicemia of catfish (ESC), while blue catfish are highly resistant against ESC disease but susceptible to columnaris disease [4]. Channel catfish and blue catfish are also useful research models for morphological, developmental, and environmental studies. They share a similar morphology, but exhibit sharp differences in body color, anal fin structure, and head size: channel catfish is light brown in color with pigmented spots on its body, while blue catfish is silver-blue in color and body spots are rare; channel catfish has fewer than 28 anal fin rays while blue catfish has more than 30 anal fin rays; channel catfish has a broader head while blue catfish has a smaller head compared to body size [4]. In nature, channel catfish rarely grow to more than 30 pounds, while blue catfish can grow to over 100 pounds; channel catfish habituate at the bottom of water columns while blue catfish habituate in the middle of water columns, reflecting their differences in tolerance to low water oxygen and adaptation to visible light [4].

While channel catfish is cultured more than blue catfish, their F_1_ hybrid produced from mating female channel catfish and male blue catfish (CXB hybrid) exhibits a high level of heterosis in growth rate, but the reciprocal F_1_ hybrid produced from mating female blue catfish and male channel catfish (BXC hybrid) does not [5]. Because of the superior growth performance of the CXB F_1_ hybrid, it is now the predominant genotype used in the US aquaculture industry, but artificial spawning must be conducted to produce the hybrid because of the reproductive isolation of the parent species. Thus, channel catfish and blue catfish are also a useful animal model to study heterosis and speciation. Understanding their genomes would also facilitate the development of superior brood stocks for aquaculture and support their sustainability for fisheries. The fertility of the female CXB F_1_ hybrids is extremely low. However, backcrossing of the male F_1_ CXB hybrid with female channel catfish is reasonably productive. In fact, successive generations of backcross progenies can be successfully produced, and the fourth generation of backcross fish can mate naturally in aquaculture ponds [6, 7], suggesting their reproductive isolation had been overcome. Such high levels of genetic similarity between reproductively isolated sibling species provide an excellent model to determine the genomic basis for speciation.

Chromosomal rearrangements are broadly considered important for speciation because it can disrupt meiosis in heterozygous hybrids, thereby causing postzygotic reproductive isolation [8–10]. Research in plants has provided concrete evidence for this hypothesis [11], but evidence from animal systems has been rare other than from fruit flies. Previous research also indicated co-localization of genes contributing to reproductive isolation within chromosomal inversions [10]. In addition, greater genetic divergence was observed with fixed chromosomal inversions than in collinear regions of the genome [9], supporting the hypothesis that gene flows are prohibited with pericentric inversions. The genetic basis of reproductive isolation between channel catfish and blue catfish is unknown, but comparative analysis of their genomic architecture should provide insights. In the present research, we sequenced and assembled chromosomal reference genome sequences for both channel catfish and blue catfish, using PacBio long reads for framework contig construction, paired-end Illumina sequencing for consensus correction, optical mapping for contig scaffolding, and high-density genetic linkage mapping for validation of chromosome structure. The highly contiguous and accurate genome assemblies permitted comparative analysis of genome architecture, coding capacities, and genomic incompatibilities. Here we report major chromosome inversions on three different chromosomes between channel catfish and blue catfish, their genomic architecture, coding capacities and characteristics, repetitive elements, characteristics of their centromere and telomeres, and specific expansion of gene families related to their biological characteristics.

## Results

### Sequencing and assembly of the channel catfish and blue catfish genomes

We present a highly contiguous, chromosome-scale reference genome for each of the sibling species blue catfish and channel catfish, with an average nucleotide conservation of 94.5% between the species. A doubled haploid, homozygous individual served as the reference individual for each species [12], and the channel catfish was the same individual used for the generation of the Coco_1.2 genome assembly [13]. The channel catfish donor genome was sequenced to a depth of 75X with PacBio contiguous long-read (CLR) data and 48X Illumina data (Additional file 1: Table S1), and the channel catfish optical map was produced from 233X coverage of molecules filtered to a minimal length of 200 kb (Additional file 1: Table S2). The blue catfish donor genome was sequenced to a depth of 93X with PacBio (CLR) sequences and a depth of 77X with Illumina sequence; the blue catfish optical map was produced from 306X genome coverage of molecules filtered to a minimal length of 200 kb. Assembled contigs were scaffolded with the optical maps and further scaffolded by alignment with high-resolution genetic maps.

**Fig. 1:**
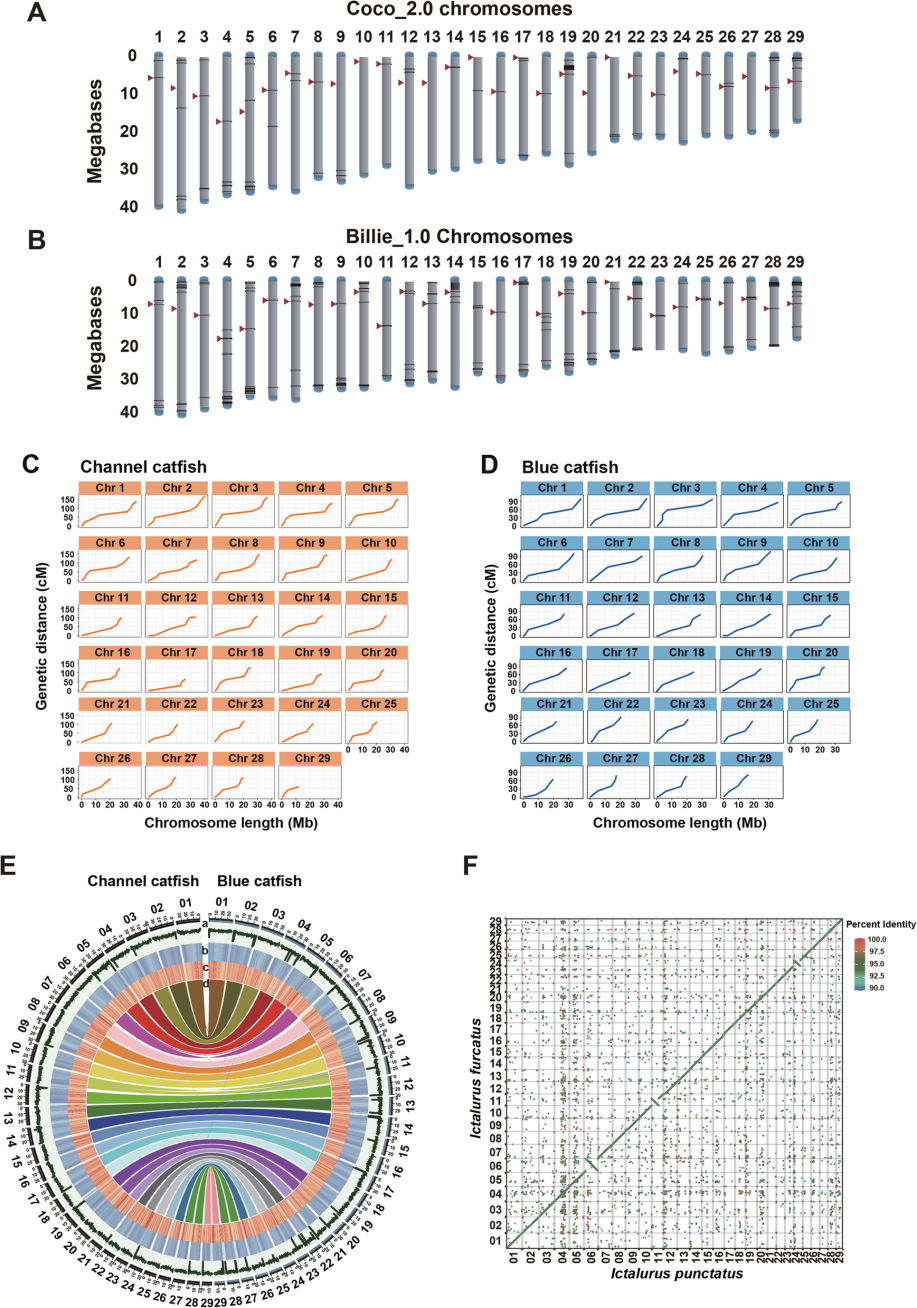
Presentation of the reference genome assemblies. **A** Channel catfish (*Ictalurus punctatus*) assembly Coco_2.0 with 29 chromosomes: Centromere positions are denoted by red triangles, telomere presence is denoted by a blue cap, and sequence gaps are denoted by black lines. **B** Blue catfish (*I. furcatus*) assembly Billie_1.0 with 29 chromosomes; annotated as above. The chromosome length is scaled in megabases. For both **A** and **B**, chromosomes are presented with the centromeres in the upper half of the chromosomes, including those of chromosomes 6, 11, and 24 where pericentric inversions are present between the two species. Concordance of marker positions on the genome sequences and genetic maps of channel catfish (**C**) and blue catfish (**D**) are presented as plot of chromosome physical length (*x*-axis) versus genetic distance (*y*-axis). **E** Circos presentation of the linear relationships between the channel catfish and blue catfish genomes, with GC content (track a), repeat elements density (track b), gene density (track c), and the collinearity of protein-coding genes (track d). **F** Dot plot of MUMmer whole-genome sequence alignments of the channel catfish (*x*-axis) versus blue catfish (*y*-axis) chromosomes

Both reference assemblies of channel catfish and blue catfish each contain 29 chromosomes, equal to their karyotype (Fig. 1A, B). The channel catfish chromosome assembly, Coco_2.0, includes 814 Mb in 96 contigs, while the blue catfish chromosome assembly, Billie_1.0, includes 815 Mb in 168 contigs (Table 1). There are 67 gaps within the channel catfish assembled chromosomes, 17 of which are within the repetitive satellite DNA of centromeres (Fig. 1A; Additional file 1: Table S3). Similarly, there are 139 gaps within the blue catfish assembled chromosomes, 22 of which are within the repetitive satellite DNA of centromeres (Fig. 1B; Additional file 1: Table S3). Most gaps are relatively small, while larger gaps involve repetitive sequences of tandem arrays such as rRNA gene clusters or within centromeric or telomeric regions in both genome assemblies.

**Table 1:** Summary and comparison of genome assembles and annotation of channel catfish and blue catfish

	Blue catfish (Billie_1.0)	Channel catfish (Coco_2.0)	Channel catfish (Coco_1.2)
**Chromosomal scaffolds**^**a**^			
Total assembled sequence (bp)	825,459,183	842,926,452	783,193,925
Bases in chromosomes (bp)	815,116,898	814,096,688	758,102,267
Scaffold NG50 (bp)	30,432,001	29,208,522	26,699,778
Bases in unassigned contigs (bp)	10,342,285	28,829,764	25,091,658
Sequence in chromosomes (%)	98.7	96.6	96.8
Protein-coding genes	23,546	25,035	23,100^b^
GC content (%)	38.95	39.71	39.70
Repetitive sequence (%)	45.5	47.6	44.0
Chromosomal scaffolds	29	29	29
Number of contigs	168	96	24,110
Number of gaps	139	67	24,080
**Sequence scaffolds**^**a**^			
Number of sequence scaffolds	664	454	9974
Sequence scaffold L50	15	17	31
Sequence scaffold N50 (bp)	23,836,692	19,747,932	7,726,806
Sequence scaffold L90	39	48	185
Sequence scaffold N90 (bp)	6,695,992	5,219,235	498,561
Number of gaps	406	211	24,109
**Sequence contigs**			
Number of contigs	1070	645	34,615
Contig NG50	6,717,455	12,841,013	69,181
Contig L50	34	22	2,839
Contig L90	206	102	10,984
Contig N50 (bp)	6,717,455	12,841,013	77,200
Contig N90 (bp)	522,535	1,003,721	16,103
Longest contig (bp)	23,646,617	29,470,913	607,423
**Centromeres**			
Centromeres identified	29	29	-
Fully sequence centromeres	7	9	-
Partial centromere sequenced	22	20	-
**Telomeres**			
Sequenced from chromosome	29	29	-
Sequenced from both ends	19	22	-
Sequenced from 5′ end	2	0	-
Sequenced from 3′ end	8	7	-

^a^Chromosomal scaffolds were built from sequence scaffolds and genetic maps. Sequence scaffolds were built from PacBio sequence contigs and Optical maps for Billie_1.0 and Coco_2.0, whereas Coco_1.2 scaffolds were built from Illumina contigs and mate-paired reads

^b^Number of genes annotated by NCBI

The reference genome assemblies are close to complete, representing 96.6% of the channel catfish genomic sequence (base pair quality value 37) and 98.7% of blue catfish genomic sequence (base pair quality value 39), respectively. There is more unassigned sequence (28.8 Mb) in the channel assembly than in the blue catfish assembly (10.3 Mb), presumably due to a greater fraction of repetitive elements in the genome of channel catfish (47.6%) than in blue catfish (45.5%) (Table 1).

The channel catfish Coco_2.0 assembly contains 59 Mb (7.8%) more sequence than the previous Illumina-based sequence assembly (Coco_1.2). Every chromosome was longer with a significant fraction of the newly assembled additional sequences being repetitive elements (Additional file 1: Fig. S1). Most striking is the decrease in the number of gaps from 24,080 in Coco_1.2 to 67 in Coco_2.0, and the new assembly included both centromeric and telomeric sequences.

The accuracy of the reference genome assemblies was enhanced using independent methodologies. Assembled sequence contigs were scaffolded by integration into optical maps which mainly produced chromosome arm scaffolds. The integration of scaffolds into each respective genetic linkage map produced full-length chromosomes. The marker positions were fully concordant with the genome sequences (Fig. 1C, D) indicating the assemblies accurately represented the chromosomes. The relationship between genetic distance and chromosomal position was not entirely linear because of the lack of recombination around centromeric regions on the linkage maps of blue catfish (our unpublished data) and channel catfish [14–16]. The sequences of the 29 chromosomes of channel catfish and blue catfish genomes were highly co-linear, as demonstrated by collinearity of protein-coding genes (Fig. 1E) and sequence alignment (Fig. 1F), with exceptions of major chromosomal inversions as described below.

### Chromosomal inversions and structural variations

**Fig. 2:**
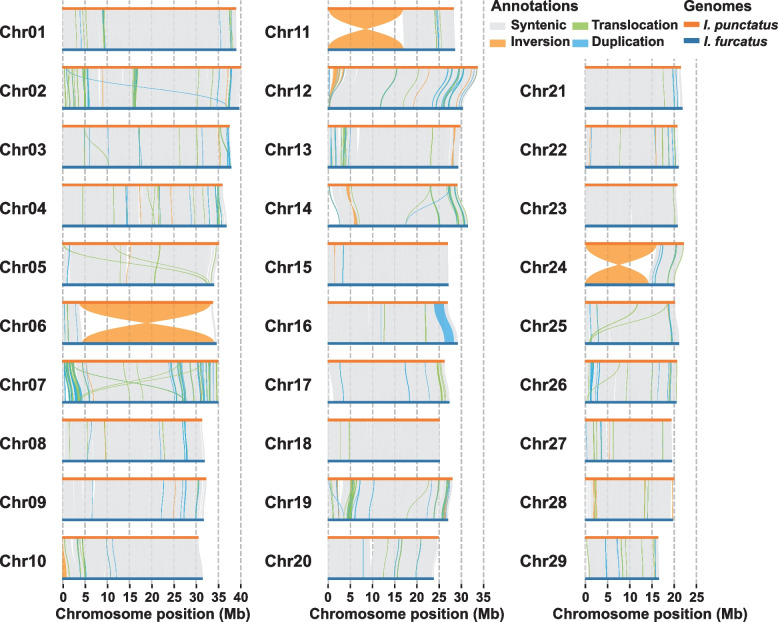
Structural variations (SVs) between the channel catfish and blue catfish genomes. Channel catfish chromosomes are orange lines and blue catfish chromosomes are blue lines. Major inversions are evident in chromosomes 6, 11, and 24

The genomic sequences of channel catfish and blue catfish were compared to determine structural variations (SVs). Using Coco_2.0 as the reference, we identified 29,593 SVs ≥ 500 bp in the Billie_1.0 assembly (Fig. 2), comprised of 2435 insertions, 1838 deletions, 179 inversions, 3413 translocations, 17,592 duplications, and other complicated variations (Additional file 1: Table S4). Four SVs exceeded 1 Mb in size; these included three large chromosomal inversions and one large segmental duplication. The three large inversions are pericentric, on chromosomes 6, 11, and 24. The inversion on chromosome 6 was the largest, involving 29.81 Mb of blue catfish sequence and 29.66 Mb of channel catfish sequence. The inversion on chromosome 11 involved 17.0 Mb of blue catfish sequence and 16.70 Mb of channel catfish sequence. The inversion on chromosome 24 involved 14.25 Mb of blue catfish sequence and 15.97 Mb of channel catfish sequence (Additional file 1: Table S5). These inversions have been confirmed in independent blue and channel haplotype assemblies produced from F1 hybrid genomic DNA (data not shown). While these major inversions caused chromosomal structural changes, the number and content of genes involved in the inversional segments were very similar between the two species (Additional file 1: Table S6). In contrast, the structural variation on chromosome 16 involved segmental duplication of 2.1 Mb (Additional file 1: Table S5), and it represented a structural variation between individuals, detected in the Billie genome but not in other blue catfish genomic templates.

**Fig. 3:**
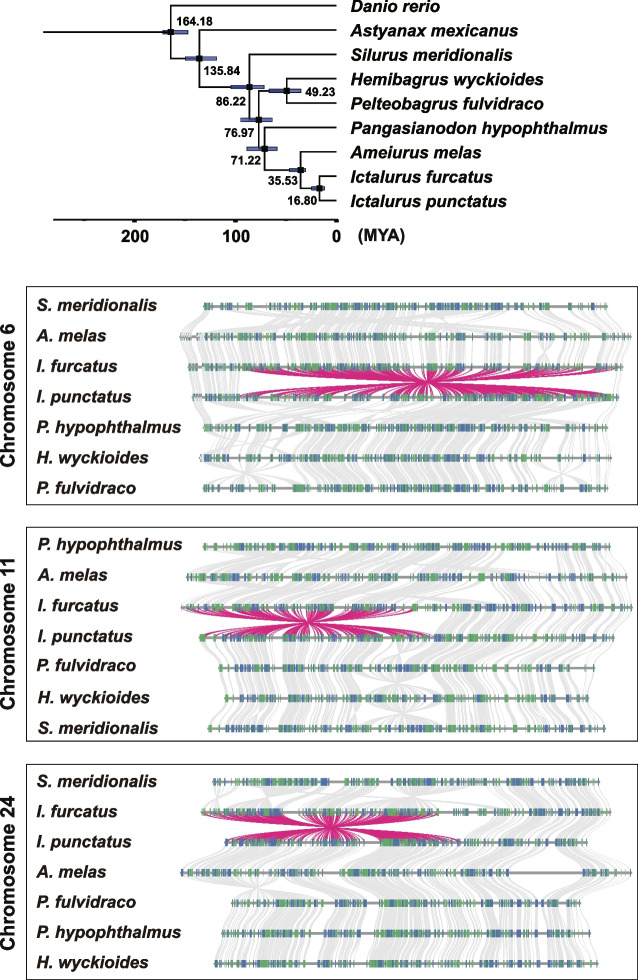
Collinearity analysis of the pericentric inversions observed between blue catfish (*Ictalurus furcatus*) and channel catfish (*I. punctatus*). Collinearity syntenic analysis of the three pericentric inversions. **A** Phylogenetic dendrogram of the species involved in the analysis. Asian species include Southern catfish (*Silurus meridionalis*), Asian redtail catfish (*Mehibagrus wyckioides*), Yellow catfish (*Pelteobagrus fulvidraco*), and striped catfish (*Pangasianodon hypophthalmus*). North American species include black bullhead catfish (*Ameiurus melas*), blue catfish *(Ictalurus furcatus*), and channel catfish (*I. punctatus*). Zebrafish (*Danio rerio*) and Mexican tetra (*Astyanax mexicanus*) are included as outgroups. Divergence times are in million years ago. **B–D** Collinearity analyses of the inversions in chromosome 6 (**B**), chromosome 11 (**C**), and chromosome 24 (**D**)

We conducted gene-based syntenic analysis [17] to determine if such pericentric inversions are present in related catfish species and if they are related to phylogenies (Fig. 3A). It appeared that similar inversions had also occurred in a number of catfish species although the exact break points varied among the species. With chromosome 6, the orientation of the inversional segment was shared among blue catfish, black bullhead (*Ameiurus melas*), and southern catfish (*S. meridionalis*), while the inverted orientation was observed with channel catfish, iridescent shark catfish (*P. hypophthalmus*), yellow catfish (*P. fulvidraco*), and redtail catfish (*H. wychioides*) (Fig. 3B). With chromosome 11, blue catfish, black bullhead, and iridescent shark catfish shared the same orientation of the inversional segment, while the remaining four species shared the other orientation of the inversional segments (Fig. 3C). For chromosome 24, only blue catfish and southern catfish shared the same orientation, while all the other five species shared the other orientation of the inversional segments (Fig. 3D). These results suggested that the pericentric inversions on chromosomes 6, 11, and 24 occurred broadly among catfish species, and they occurred independently in these taxa.

### Molecular and genetic evidence of the chromosomal inversions

**Fig. 4:**
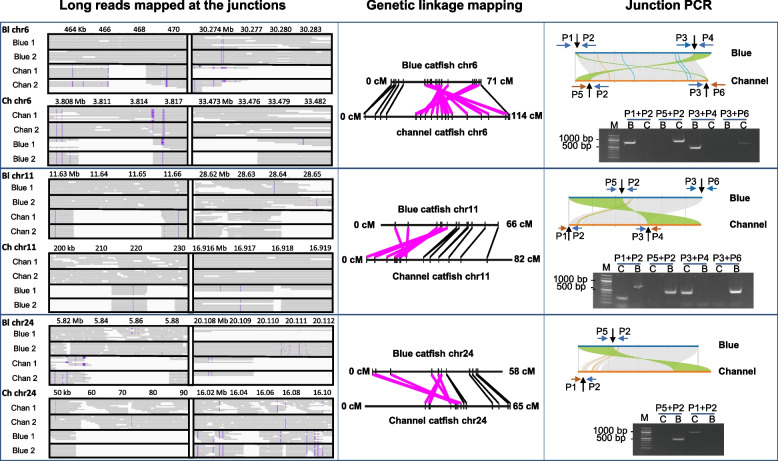
Evidence of pericentric inversions between the genomes of channel catfish and blue catfish. Three lines of evidence supported the presence of major pericentric inversions on chromosome 6, chromosome 11, and chromosome 24: (1) long reads mapped at the junctions (left panel); (2) genetic linkage mapping (middle panel); and (3) junction PCR (right panel). With long reads across the inversional junctions, two additional blue catfish and two channel catfish were sequenced in addition to the sequencing templates that were used to generate the reference genomes. Alignments were contiguous when the junctional long reads from blue catfish individuals were mapped against the reference genome sequence of blue catfish, but not against the reference genome sequence of channel catfish and vice versa. This was true for all inverted chromosomes 6, 11, and 24, and for both the left and the right inversion junctions. With genetic mapping, a common set of markers (Additional file 1: Table S17) were identified within the inverted junctions, and the inversion was evident for all inverted chromosomes of 6, 11, and 24. Finally, we designed PCR primers to amplify across the inversion junctions (Additional File 1: Table S17). As expected, the PCR amplicon matched the expectation of inversions, except for the left junction PCR using primers of channel catfish, which generated a band from blue catfish as well, but not of expected size. We believe this band was generated from non-specific primer binding as an artifact

Analysis of reference genome sequences revealed the three pericentric inversions on chromosomes 6, 11, and 24. To demonstrate and validate the pericentric inversions, we took three different approaches: (1) analysis of long sequencing reads across the inversion junctions from multiple individuals; (2) genetic linkage analysis of a common set of markers that can be mapped to both blue catfish and channel catfish; and (3) PCR analysis across the inversion junctions. With analysis of long reads across inversion junctions, we independently mapped long reads from two blue catfish and two channel catfish unrelated to the reference genomes. As shown in Fig. 4, in all cases, long reads across the inversion junctions from both blue catfish aligned well with the reference genome of blue catfish Billie_1.0, but not with the reference genome of channel catfish; similarly, in all cases, long reads across the inversion junctions from both channel catfish aligned well with the reference genome of channel catfish Coco_2.0, but not with reference genome of blue catfish, providing additional sequence support for the accurate assemblies of the reference genomes of blue catfish and channel catfish. With the second approach, genetic linkage analysis was conducted using a common set of markers (Additional file 2: Table S7) that can be mapped to both blue catfish and channel catfish. As shown in Fig. 4, common markers were mapped to opposite locations within the inverted segments, providing genetic evidence for the pericentric inversions. With the third approach, PCR primers were designed flanking five of the six inversion junctions for both blue catfish and channel catfish (one primer set on chromosome 24 could not be designed uniquely because the flanking sequence consisted of telomeric repeats). As shown in Fig. 4, PCR amplicons were produced as expected of the pericentric inversions, providing molecular support for the pericentric inversions.

**Fig. 5:**
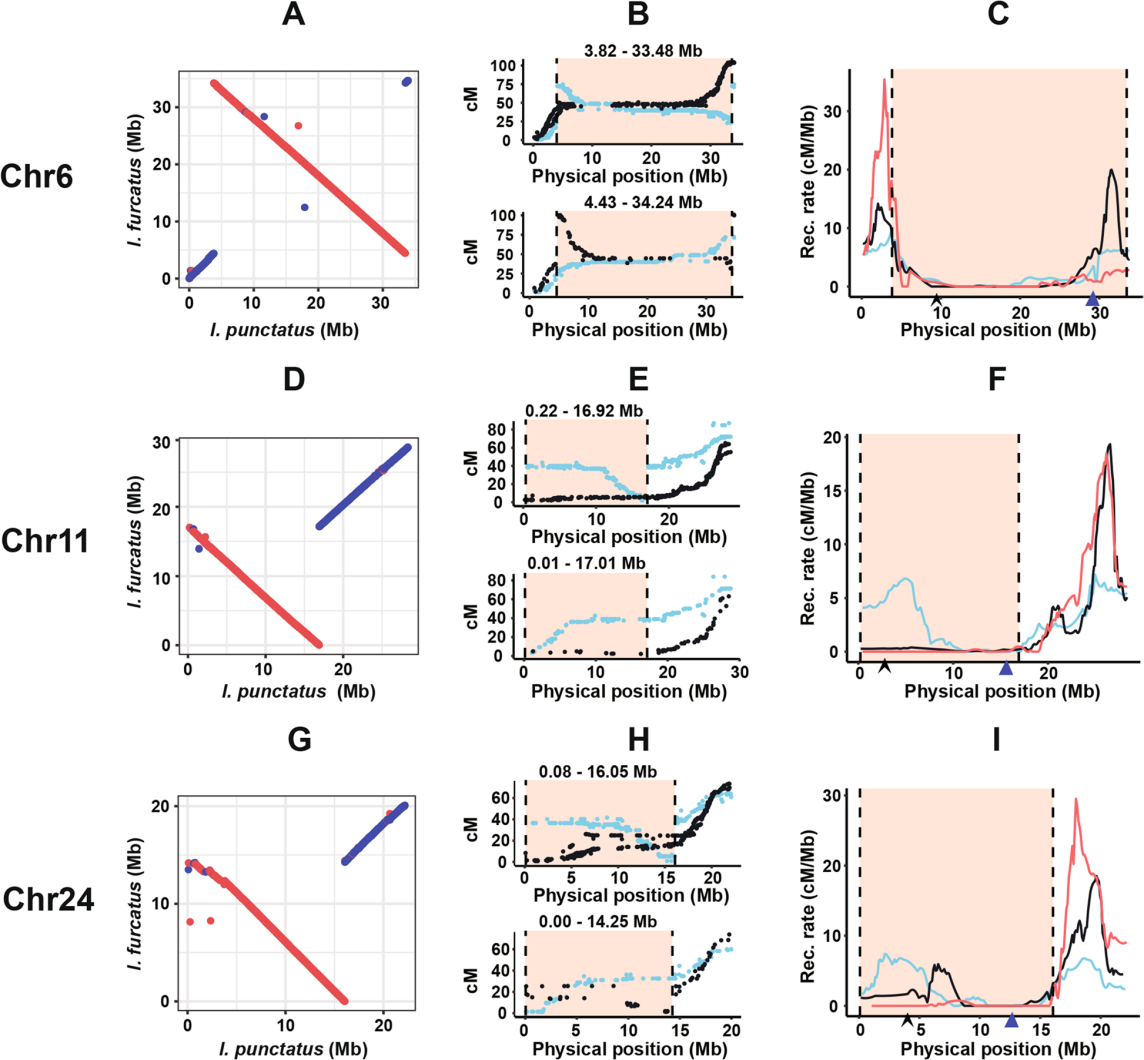
Recombination rates within pericentric inversions. Left panel: Dot plots of MUMmer alignments of channel catfish and blue catfish chromosome 6 (**A**), chromosome 11 (**D**), and chromosome 24 (**G**) are presented, all using reverse complement sequences of blue catfish as deposited in NCBI. Middle panel: Plot of genetic positions (*y*-axis) against physical positions (*x*-axis) of markers on Coco_2.0 (upper) and Billie_1.0 (lower) for chromosome 6 (**B**), chromosome 11 (**E**), and chromosome 24 (**H**). The orange background denotes the boundaries of the chromosomal inversions. Right panel: Plot of recombination rates (cM per Mb, *y*-axis) vs physical positions (Mb) of genetic markers on the genomic sequences for chromosome 6 (**C**), chromosome 11 (**F**), and chromosome 24 (**I**), with recombination rates of channel catfish indicated in black, blue catfish in blue, and hybrid catfish in red. The black triangle and blue triangle indicate the position of the centromere in channel catfish and blue catfish, respectively

The major pericentric inversions on chromosomes 6, 11, and 24 imply that gene flow between channel catfish and blue catfish could be restricted on these chromosomes. If so, the recombination rates would be lower in the inversional segments on these chromosomes in the gametes of F_1_ hybrid (channel catfish × blue catfish). We determined the recombination rates using genetic linkage analysis. Recombination rates were calculated from genetic linkage mapping using channel catfish intraspecific resource families [14]; those for blue catfish were calculated from genetic linkage mapping using blue catfish intraspecific families (unpublished), and those for hybrids were calculated from genetic linkage mapping using interspecific backcross progenies, where channel catfish were mated with interspecific F_1_ hybrid (channel catfish female × blue catfish male) to produce the backcross progenies [15]. As shown in Fig. 5, there was no recombination in the interspecific hybrid within the inversional segments, other than very low numbers of double crossovers, as predicted. In contrast, there were recombination events within the inversional segments with channel catfish or blue catfish. Significantly higher recombination rates were observed within channel catfish or blue catfish than in backcross progenies, despite the overall low recombination rates surrounding the centromeres (Fig. 5), suggesting postzygotic inhibition of recombination or mortalities of the recombinants.

### Genome annotation and protein-coding capacities

To annotate the channel and blue protein-coding genes, we combined results obtained from protein-homology-based prediction, RNA-seq-based prediction, and Breaker2 prediction. A total of 25,035 high-confidence protein-coding genes were predicted in the channel catfish genome, of which 24,558 (98.1%) genes were included in the 29 chromosomes (Additional file 1: Table S8). The total number of protein-coding genes was increased by 1935 from Coco_1.2, and the number of protein-coding genes unassigned to chromosomes was decreased from 823 to 477. Similarly, a total of 23,546 high-confidence protein-coding genes were predicted in the blue catfish genome, of which 23,444 genes (99.6%) were included in the 29 chromosomes; only 102 blue catfish protein-coding genes were unassigned to chromosomes (Additional file 1: Table S8).

**Fig. 6:**
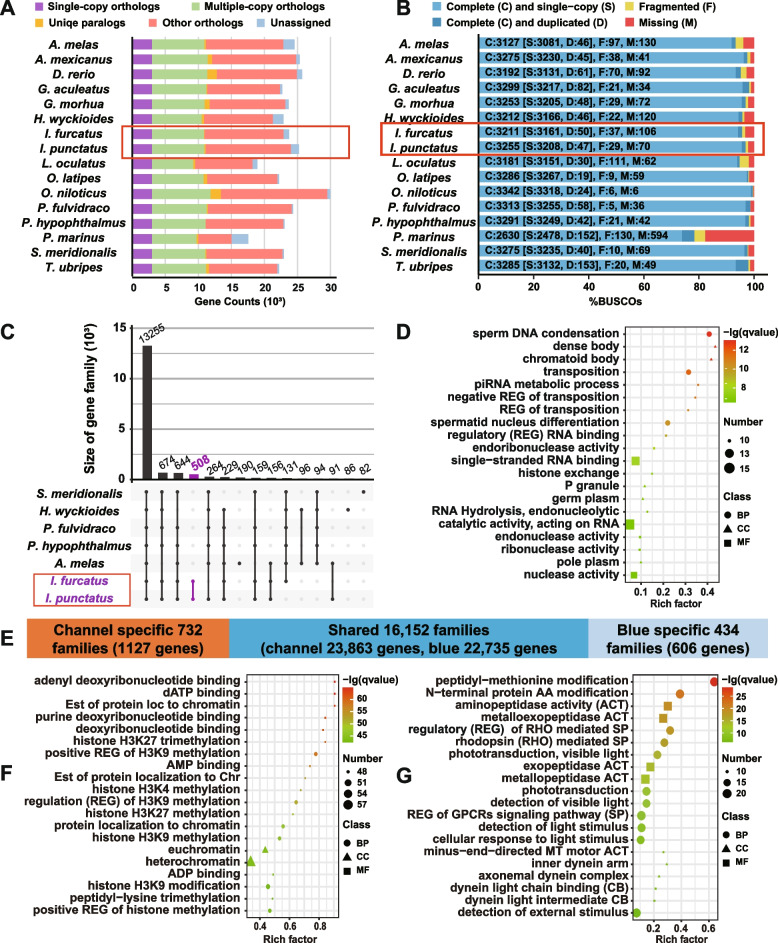
Annotation of the channel catfish and blue catfish genomes. **A** Comparison of sequence orthology between 16 fish species showing number of genes in each ortholog category, with channel catfish and blue catfish highlighted in the red box. **B** Comparison of the 3640 Actinopterygii (ray-finned fish) BUSCO genes among the genome assemblies of 16 fish species. **C** Analysis of distinctive genes (or gene families) among seven catfish species whose genome has been sequenced. Channel catfish and blue catfish are highlighted in the red box, and genes specific to them are highlighted in purple. **D** Enrichment analysis of genes specific to channel catfish and blue catfish. **E** Summary of the commonality and difference of channel catfish and blue catfish genes. **F** Enrichment analysis of genes specific to channel catfish. **G** Enrichment analysis of genes specific to blue catfish

The numbers of protein-coding genes identified from the channel catfish and blue catfish genomes compare favorably with their orthologous counterparts from well assembled fish species (Fig. 6A; Additional file 1: Table S9). Of the 3640 Actinopterygii (ray-finned fish) BUSCO genes [18], 3517 (96.6%) and 3480 (95.6%) were detected in the channel catfish genome Coco_2.0 and blue catfish genome Billie_1.0, respectively, as compared to 3475 (95.47%) in the *Danio rerio* genome (Fig. 6B).

We also compared the distinct protein-coding gene families of the seven catfish genomes for which whole-genome sequences are available, including southern catfish (*Silurus meridionalis*) [19, 20], Asian redtail catfish (*Hemibagrus wyckioides*) [21], yellow catfish (*Pelteobagrus fulvidraco*) [22], striped catfish (*Pangasianodon hypophthalmus*) [23], black bullhead catfish (*A. melas*, GenBank Accession GCA_012411375.1), and channel catfish and blue catfish. A total of 18,320 gene families were inferred from the seven catfishes, of which 13,255 gene families were shared by all seven catfish species. The remaining 5095 gene families were shared by a variable number of 1 to 6 catfish species (Fig. 6C, displaying only the top 15 shared groups of gene families). Of particular interest were the 508 gene families that were specific to the ictalurid channel catfish and blue catfish (Fig. 6C; Additional file 2: Table S10). Enrichment analysis indicated that the 508 channels and blue catfish-specific gene families were enriched for functions related to spermatid development, negative regulation of transposition, and RNA hydrolysis (Fig. 6D).

Comparison of the protein-coding capacities between channel catfish and blue catfish revealed 732 gene families specific to channel catfish (Additional file 2: Table S11) and 434 gene families specific to blue catfish (Additional file 2: Table S12). The channel catfish gene families included 1127 individual genes, and the blue catfish gene families included 606 individual genes (Fig. 6E). Enrichment analysis indicated that 1127 channel catfish-specific genes were enriched with chromatin structure of euchromatin and heterochromatin, especially ATP- and AMP-binding activities related to histone H3 modifications (Fig. 6F). In contrast, the 606 blue catfish-specific genes were enriched for (1) amino acid modification involving amino- and metalloexo-peptidase activities; (2) responses to light involving rhodopsin-mediated signaling pathway; (3) cellular motility-related functions involving dynein and microtubules; and (4) immune-related function involving MHC class I biosynthesis and interleukin 18 production (Fig. 6G).

### Expansion of repeatome

**Fig. 7:**
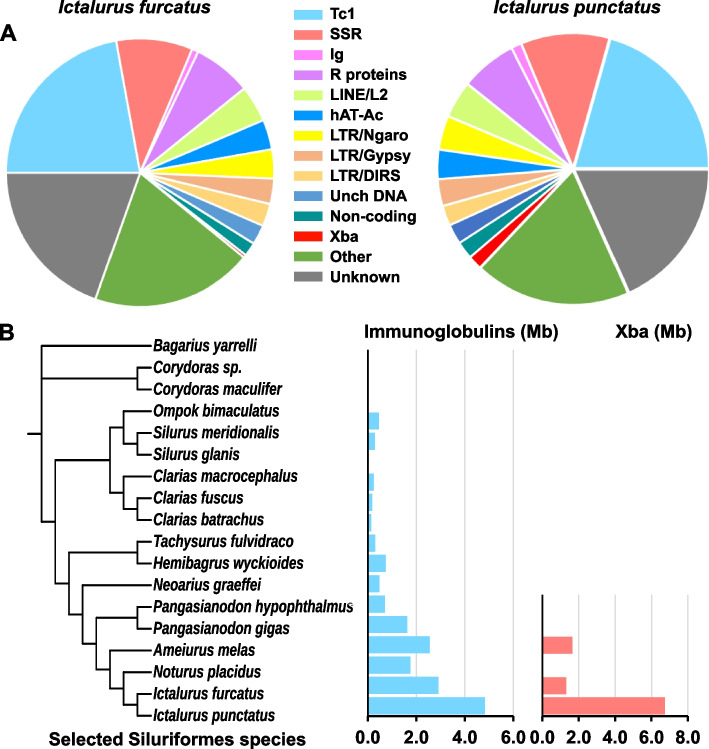
The repeatomes of channel catfish and blue catfish and their specific expansion of immunoglobulin-related genes and the Xba elements. Repeatomes of channel catfish and blue catfish. **A** The most abundant categories of the repeatomes of blue catfish (left) and channel catfish (right), with each category representing at least 1% of the repetitive elements of their repeatome, respectively. The complete list and annotation of their repetitive elements are presented in Additional file 1: Table S14. **B** Distribution and quantity (in Mb) of immunoglobulin-related proteins (Immunoglobulins) and Xba elements in various catfish species. The amount in Mb of immunoglobulin-related proteins and Xba elements are indicated at the bottom of the figures. Phylogenetic analysis was conducted with cytochrome b sequences, and each bar corresponds to the species within the phylogenetic tree with immunoglobulins in blue, and Xba elements in orange

The blue catfish and the channel catfish genome assemblies contain 45.5 and 47.6% repetitive elements, respectively. The top 17 categories of repetitive elements (representing at least 1% of the repeatome) in blue catfish accounted for 66.2% of all repetitive elements in the blue catfish genome (Fig. 7A, Additional file 2: Table S11), with Tc1/mariner transposons (22.1%) most abundant followed by simple sequence repeats (9.2%), repetitive proteins (7.8%), LINE/L2 (4.4%), DNA/hAT-Ac (3.6%), LTR/Ngaro (3.5%), LTR/Gypsy (3.1%), LTR/DIRS (2.7%), uncharacterized DNA transposon (2.4%), Satellite (1.5%), LTR/ERV1 (1.4%), LINE/Rex-Babar (1.2%), Repetitive non-coding RNA (1.7%), DNA/PIF-Harbinger (1.4%), DNA/CMC-EnSpm (1.4%), DNA/hAT-Charlie (1.4%), and LINE/Rex-Babar (1.2%). The remaining 50 categories of known repetitive elements accounted for 14.2% of the repeatome of blue catfish. A total of 19.6% of repetitive elements are unknown in nature (Additional file 1: Table S13).

The categories and proportions of the repetitive elements in the channel catfish genome are similar to those in the blue catfish genome [24], with exception of the Xba elements and immunoglobulin-related repetitive proteins (Additional file 1: Table S14). The channel catfish genome contains significantly more Xba elements than the blue catfish genome, accounting for 1.7% of its repeatome, as compared to 0.35% in blue catfish. The Xba elements are centromeric (see below), but another major repeatome expansion of channel catfish and blue catfish is repetitive proteins, accounting for almost 8% of their repeatome (approximately 4% of the genome). In particular, the immunoglobulin-related genes are significantly expanded in the catfishes (Siluriformes) compared to other teleost and vertebrate taxa. Immunoglobulin-related gene sequences comprise 4.8 Mb (channel catfish) and 2.9 Mb (blue catfish) of the genome (Fig. 7B). The amount of immunoglobulin-related gene sequences in the genomes of various catfishes exhibited an interesting pattern. Of the 18 species analyzed, channel catfish has the largest proportion of immunoglobulin-related gene sequences in its genome, followed by blue catfish, black bullhead (*A. melas*), Neosho madtom (*N. placidus*), and Giant Mekong catfish (*Pangasianodon gigas*), while immunoglobulin-related gene content declined in proportion to the phylogenetic distance from ictalurid catfishes (Fig. 7B).

### Centromeres and telomeres

**Fig. 8:**
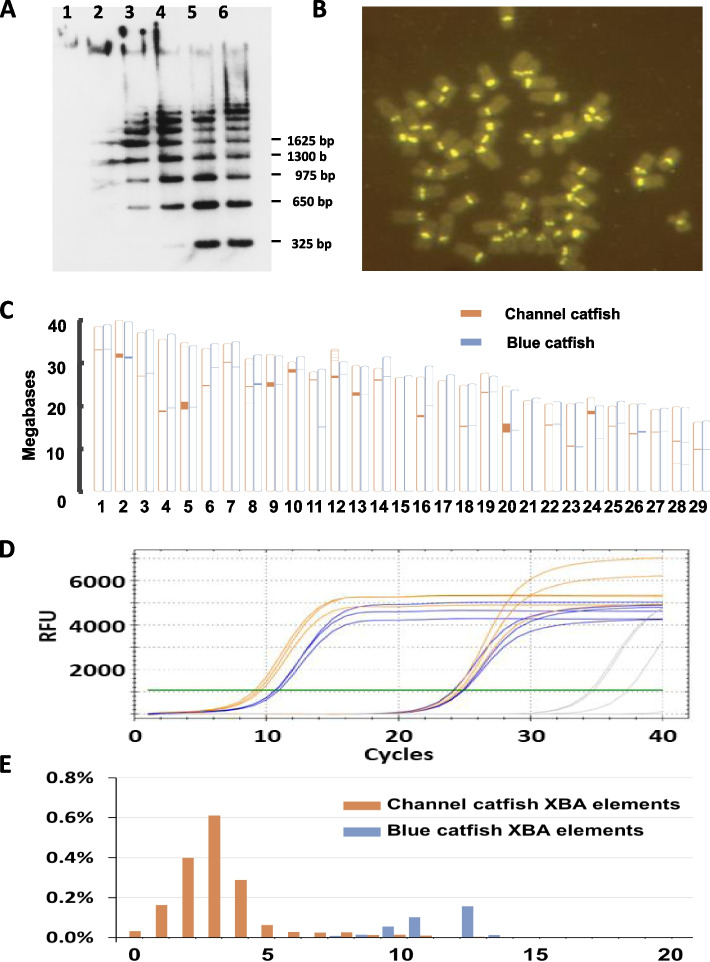
Analysis of centromeres of channel catfish and blue catfish. **A** Southern blot analysis of channel catfish genomic DNA digested with Xba I restriction endonuclease (adopted from [25]), showing tandem structure as a ladder was produced with incremental amounts of Xba I enzyme (Lanes 1–6). The molecular weight standards are indicated on the right margin. **B** Fluorescence in situ hybridization of Xba elements labeled with digoxigenin and detected with FITC-labeled anti-digoxigenin on channel catfish metaphase chromosomes (2*n* = 58), adopted from Quiniou et al. [26]. **C** Comparison of relative centromeric locations and sizes of channel catfish (orange) and blue catfish (blue) chromosome scaffolds. Note that the relative size of centromeres was amplified by × 2 to clearly show the difference between channel catfish and blue catfish. **D** Quantitative real-time PCR using Xba element-specific primers. The Ct values of 8.96 10.43, and 24.63 were observed for channel catfish Xba (orange), blue catfish Xba (blue), and a single-copy microsatellite marker (gray), respectively. **E** Divergence rates of Xba elements of channel catfish (orange) and blue catfish (blue) as denoted by the Xba elements as a percentage of total genomic repeats versus substitution rate (% substitution per site)

The genome assemblies permitted characterization of centromeric and telomeric repeats in channel catfish and blue catfish. The centromeres of channel catfish and blue catfish are composed of satellite sequences of Xba elements [25, 26]. The Xba elements are highly AT-rich (65.5%); the vast majority are 321–325 bp long organized in head-to-tail tandem arrays in the centromeres (Fig. 8A, B). Relative centromere positions were also conserved between channel catfish and blue catfish except for chromosomes 6, 11, and 24 due to the inversions (Fig. 8C; Additional file 1: Table S15). Gene synteny surrounding the centromeres on these three chromosomes was entirely conserved.

The numbers of Xba tandem repeats varied greatly among chromosomes of both species (Additional file 1: Table S16). All chromosomes in the Coco_2.0 assembly contained centromeres, with nine chromosomes containing ungapped centromeres. The largest ungapped centromere contained 3146 Xba repeat units (chromosome 20) with a total length of over one million base pairs. Similarly, centromeres were identified in all but one blue catfish assembled chromosome, seven contained ungapped centromeres, the largest of which contained 635 Xba repeat units. While ungapped assembly of Illumina-corrected CLR sequence may not necessarily equate to complete centromeres, the genomic sequence pointed to larger centromere sizes in channel catfish than in blue catfish (Fig. 8C). Therefore, we validated the size difference using real-time quantitative PCR on genomic DNA from unrelated channel and blue catfish and found 2.25-fold more centromeric DNA in the channel catfish genome than in the blue catfish genome (Fig. 8D).

The sequences of the Xba elements are highly conserved, with the highest levels of conservation among Xba elements within a single centromere of channel catfish. The sequences of Xba elements are significantly more divergent in blue catfish (Fig. 8E), even among repeat units within a single centromere (Additional file 1: Fig. S2). The average percent of substitution rate of Xba elements in channel catfish was 3.1, whereas that of Xba elements in blue catfish was 11.7, almost four times larger. In both channel catfish and blue catfish, the Xba element sequence varied more at the beginning and end of each centromere, and sequences of the internal repeat units were most highly conserved.

Despite high levels of sequence conservation, centromeric Xba sequences appear to be present only in some species within Ictaluridae. We searched catfish whole-genome sequences in GenBank for Xba elements. In addition to channel catfish and blue catfish, Xba elements were present in the Ictalurids *A. melas* and *N. placidus* but not in any other organisms, including various catfish species (Fig. 7B). We do not know if the Xba elements serve as centromeres in *A. melas* as they do in channel catfish and blue catfish, but their distribution in various chromosomes in the form of tandem repeats suggests that role. However, copy numbers of Xba elements in *N. placidus* are very low—either this is an artifact of the sequencing platform or Xba elements may not be centromeric in this species.

Telomeric sequences were identified in all 29 chromosomes of channel catfish, of which TTAGGG repeats were identified both at the beginning and at the end in 22 chromosomes (Additional file 1: Table S15). For the 7 remaining chromosomes (2, 3, 10, 11, 15, 17, and 21), telomeric repeats present in the unlocated sequencing data could not be uniquely placed at the p-arm of the chromosomes. Three of the latter chromosomes (15, 17, 21) were acrocentric or telocentric based on optical mapping data (Additional file 1: Table S15). Similarly, for blue catfish, telomeric TTAGGG repeats were present at both ends of 19 chromosomes. Again, the p-arm sequence began with centromeric Xba elements in the acrocentric/telocentric chromosomes 17 and 21. Centromeric sequences were missing from the p-arm of chromosome 15. Five additional chromosomes did not have telomeric sequence on the p-arm and two did not have telomere sequence on the q-arm (Additional file 1: Table S15).

There are some variations of telomere repeats with channel catfish. For example, its chromosome 12 has a long repeat sequence of 102 bp which appeared to have a higher order of repeat (HOR) (GGGCTTCCCCAGGCTCGGTGAGTGATTTTCGGGCAAAATGACAAACTTCCACAGGCGTTTCCCTTGAACCGAGCTCCATCAGGGGCTTCAGTACT/GGGTTA); chromosome 13 has a repeat sequence of AGAGGGG at the beginning but regular TTAGGG at the end; chromosome 22 has repeats of AAACAGTTAG(T/C)GATG/GGGTTA; chromosome 27 has TTAGGG on the same strand at both 5′-end and 3-end of the chromosome.

## Discussion

We report reference genome sequences of channel catfish and blue catfish. These reference genomes will be valuable resources for various biological, environmental, and evolutionary studies. We previously published a reference genome sequence for channel catfish [13] that was produced using second-generation sequencing technology. The continuity of the current Coco_2.0 assembly is drastically enhanced, from a total of 34,615 contigs and 9974 sequence scaffolds in Coco_1.2 to only 96 contigs in Coco_2.0. In addition to continuity and more repetitive content, Coco_2.0 includes 1935 more protein-coding genes compared to Coco_1.2. Much like Coco_2.0 for channel catfish, the blue catfish genome sequence is highly contiguous (Table 1).

A blue catfish genome assembly has been recently reported [27], but we believe the Billie_1.0 assembly is more robust and more accurately reflects the blue catfish genome. The three large chromosomal inversions between blue catfish and channel catfish genomes were not reported by Wang et al. [27]; another 6.8 Mb inversion, on chromosome 7 (position 20,122,920–26,935,075), may represent an artifact in their assembly (Additional file 1: Fig. S3 and Fig. S4). The Billie_1.0 assembly was produced using three independent resources—long-read sequencing and optical mapping from the D&B strain of blue catfish genome donor, and genetic mapping of three unrelated blue catfish full-sibling families derived from the Rio Grande strain or from parents collected from the Mississippi River. The markers on the genetic map and on the physical sequence are concordant. Furthermore, we have produced two additional blue catfish haploid assemblies and two channel catfish haploid assemblies derived from genomic sequences of two F_1_ hybrid individuals and all four new assemblies confirm the inversions in Billie_1.0 compared to Coco_2.0. Scaffolding of the blue catfish genome assembly by Wang et al. [27] utilized genetic linkage maps constructed for channel catfish that were derived from either channel catfish resource families or interspecific hybrid resource families [14–16], suggesting that caution should be exercised when conducting reference-guided assemblies even of closely related species.

Three lines of evidence support the inversions we report here between blue catfish and channel catfish genomes on chromosomes 6, 11, and 24 (Fig. 4). First, long reads across the inversion junctions using unrelated blue catfish and channel catfish all are compatible with the inversions. Second, genetic linkage mapping, as conducted using resource families that are unrelated to any of the multiple sequencing templates, also supported the chromosomal inversions. Third, direct test through PCR using primers across the inversion junctions also supported the chromosomal inversions, although on chromosomal 24 unique PCR primers could only be designed for the inversion junction at the beginning of the chromosome (Fig. 4). In addition, the primer pair at the beginning of chromosome 11 produced an unexpected band from blue catfish, likely from non-specific primer binding because the size was wrong even if there was no inversion.

The functional importance of the pericentric inversions in speciation of channel and blue catfish is unknown at present, because combining genomes with huge chromosomal inversions are not necessarily postzygotic barriers. Many fish species have multiple large inversions segregating in the populations (e.g., Atlantic cod) with impact on recombination but no apparent impact on hybrid fitness [28]. The observation of low recombinants among backcross progenies (Fig. 5) could indicate either lack of recombination or mortality of the recombinants. The detection of low rate of double crossover recombinants, but not single crossover recombinants, among backcross progenies suggested the latter, indicating that the pericentric inversions could be a postzygotic barrier for survival of the recombinants. In spite of being anecdotal, our previous research [7, 29] also reported low hatching and survival rates of first generation of backcross progenies (female channel catfish × male F_1_ hybrid), but increasingly higher hatching and survival rates were observed with higher generations of backcrosses, suggesting “homogenization” of chromosomes through continuous backcrossing would be an approach to effectively introgress chromosomal segments from blue catfish into fertile hybrids.

Overall quality of the channel and blue reference genome sequence assemblies was assessed with a set of standards and metrics recommended by the G10K Consortium [30]. The channel catfish genome assembly Coco_2.0 had x.y.P.Q.C. metrics of > 12. > 29.-0.37.97 and the blue catfish genome assembly had x.y.P.Q.C. metrics of > 6. > 30.-0.39.99 (Table 2). The channel catfish genome sequence was slightly more contiguous with 67 gaps than the blue catfish genome sequence with 139 gaps. However, the blue catfish genome assembly Billie_1.0 was more complete with 98.7% of the genome sequences assigned to chromosomes while 96.6% of genome sequences of channel catfish were assigned to chromosomes. Longer centromeric repeat arrays and more unlocated repetitive elements in channel catfish contributed to the 2.1% difference. However, larger gaps in the Billie_1.0 assembly, especially one on chromosome 14, were due to tandem arrays of rDNA and tRNA genes near the ends of the chromosome. Those arrays lacked polymorphic genetic markers and could not be oriented uniquely on the chromosomes. The accuracy of the reference genome sequence was demonstrated by concordance of large numbers of SNP marker positions on the reference genome sequences with those on the genetic linkage maps [14–16] (and our unpublished data). Haplotype blocks could not be assessed from the reference genome sequences because homozygous, doubled haploid sequencing templates were used for sequencing with both channel catfish and blue catfish. These high-quality genome sequences, channel catfish genome assembly Coco_2.0 and blue catfish genome assembly Billie_1.0, and assemblies from other fish species, such as zebrafish [31], cavefish [32], Atlantic salmon [33], sterlet sturgeon [34], Silver Sillago [35], half-smooth tongue sole [36], common carp [37], tilapia and related cichlids [38], and European seabass [39], will serve as long-term resources for genetic and genomic research with teleost species, which account for more than 50% of all vertebrate species.

**Table 2:** Quality assessment of the channel catfish genome assembly Coco_2.0 and blue catfish genome assembly Billie_1.0 using International Genome 10 K (G10K) Consortium metrics [30]

Assemblies	Assembly metrics	Blue catfish Billie 1.0	Channel catfish Coco 2.0
Overall quality	x.y.P.Q.C	> 6. > 30.–.39.99	> 12. > 29.–.37.97
1. Continuity	1.1. Contig NG50 (*x*)	6.7 Mb	12.8 Mb
	1.2. Scaffolds NG50 (*y*)	30.4 Mb	29.2 Mb
	1.3. Gaps per Gb	170	82
2. Structural accuracy	2.1. Reliable blocks	2.8–33.9 Mb	7.2–33.6 Mb
	2.2. False duplications	-	-
	2.3. Curation	Manual	Manual
3. Base accuracy	1.1 Base pair QV (Q)	39.17	37.15
	3.2. k-mer completeness	98.65%	98.05%
4. Haplotype phasing	1.2 Phase block NG50 (P)	-	-
5. Functional completeness	1.3 Genes	26,575	27,504
	5.2. Transcript mappability	-	-
6. Chromosome status	1.4 Assigned (C)	99%	97%
	1.5 Sex chromosome	X	X and Y^a^
	1.6 Mitochondrial genome	One complete allele, KM576102.1	One complete allele, NC_003489.1
Additional assurances	Bionano optical mapping	306X genome coverage	233X genome coverage
Additional assurances	Genetic linkage mapping	690 K SNP arrays	690 K SNP arrays

^a^Y chromosome available in GenBank from prior research

The high-quality assemblies of these two closely related species provided a more complete landscape of genome architecture, gene annotation, repetitive elements, TE insertions, and centromere and telomere sequence characteristics. Of particular interest were the 508 genes present in channel catfish and blue catfish but absent from five other catfish species. Enrichment analysis indicated overrepresentation of several categories of genes including piRNA-binding, RNA silencing, fertility-related functions, and negative regulation of transposition, suggesting the importance in catfish of piRNA-induced silencing complexes (piRISCs) in fertility and transposon silencing [40], as they are in worms, flies, and mice [41–43]. However, such speculation is based on the assumption that the reference genomes of the species under comparison are complete; we can only assess the quality of the reference genomes of blue catfish and channel catfish reported here but have no assessments for the other catfish species used in the analysis.

A large set of genes was present in the channel catfish genome but not in the blue catfish genome. Enrichment analysis indicated the major overrepresentation terms in channel catfish are genes related to chromatin structure involving histone H3 modifications such as H3-K4, H3-K9, and H3-K27 methylation. Similarly, a total of 606 genes in the blue catfish genome were not found in the channel catfish genome. Enrichment analysis indicated that the major overrepresented terms of these genes were involved in peptidase activities, responses to light involving rhodopsin signaling, cellular motility-related functions involving dynein and microtubules; and immune-related functions involving MHC class I and interleukin 18 production. We do not know what functional importance these enriched genes mean for each species, but we do know that these differences in gene contents between blue catfish and channel catfish are real because of the completeness of the reference genomes of both blue catfish and channel catfish.

The channel catfish and blue catfish genomes are characteristic of significant expansions of immunoglobulin-related genes [44], which correlate well with the phylogenetic relationship of various catfishes (Fig. 7B). Ictalurid catfishes are endemic to temperate areas in North America [45], whereas the more distantly related catfish species are distributed in tropical or subtropical South America or southern Asia. Perhaps more significantly, the taxa with significant expansion of immunoglobulin-related genes are all scaleless catfishes that could be exposed more frequently to microorganisms. Taxa with a smaller repertoire of immunoglobulin-related genes typically have protective skin structures. The body of *Bagarius* species is entirely or almost entirely covered by heavily keratinized skin superficially differentiated into unculiferous plaques or tubercles [46], while *Corydoras* catfishes have special scales made of bony dermal plates [13]. These protective skin structures, like the scales of other teleost fish, could offer more protection from direct exposure to microorganisms.

Centromeres are important for chromosome segregation during meiosis and mitosis, and telomeres are important for maintenance of chromosomal integrity and cell longevity. However, all existing catfish genome assemblies in NCBI, including those for channel catfish, yellow catfish (*Tachysurus fulvidraco*) [22], and panga catfish (*Pangasius djambal*, GenBank accession GCA_022985145.1) have entirely excluded repetitive sequences within and near centromeres, and of telomeres. The present research included drafts of most centromeres and telomeres, although some are still incomplete. We previously characterized the Xba elements [24–26] but only as unique plasmid subclones of whole-genomic DNA. The contiguous sequences reported in the current work allowed analysis of sequence arrangements of the Xba elements within the centromere on each of the 29 chromosomes. The blue catfish and channel catfish centromeres are composed of purely Xba sequences, with minimal length variations among units of Xba elements, and without any other sequences among the units of Xba elements. The continuous sequencing also provided accurate information of Xba elements in the genome and within each centromere. Using dot blot analysis, the Xba elements were assessed to represent ~ 5–6% of the channel catfish genome [25], but the current sequence information revealed that the Xba elements in the channel catfish genome accounted for just approximately 1% of the channel catfish genome. While it is possible that the dot blot analysis [25] could overestimate the Xba contents, it is also possible that the genome assemblies are still missing substantial amounts of centromeric sequence. Analysis of the Xba elements represented in each of the 29 centromeres in blue catfish and channel catfish indicated that the lengths of centromeres can vary greatly (Table S15). For example, the longest centromere of channel catfish of chromosome 5 contained over 3146 units of Xba elements, while its shortest centromere of chromosome 17 had only 67 units of Xba elements.

Extensive sequence analysis with thousands of repeat units from channel catfish, blue catfish and black bullhead catfish indicated conservation of AT-rich blocks of sequences of approximately 24–30 bp long. Almost all centromere sequences, ranging from yeast and plants to animals including humans, are AT-rich [47], indicative of functional relevance. Centromeric monomers in non-acrocentric chromosomes were reported to evolve significantly faster than those in acrocentric chromosomes in medaka [48], but we observed no difference in divergence rate among different types of chromosomes, from metacentric, submetacentric, acrocentric, and telomeric chromosomes. Rather, the centromere repetitive sequences of channel catfish had a much smaller substitution rate compared with blue catfish, suggesting that the Xba elements in channel catfish have been actively transposing more recently than in blue catfish (Fig. 8E).

## Conclusions

The present research presents highly contiguous reference genome sequences of channel catfish and blue catfish. Comparative analysis of the reference genomes revealed three major pericentric chromosome inversions involving chromosomes 6, 11, and 24. Additional analyses of long reads across the inversional junctions, linkage mapping, and junction PCR validated these pericentric chromosomal inversions. Marker segregation analysis of chromosomes 6, 11, and 24 confirmed the lack of recombination in the backcross progeny [15], suggesting that the pericentric inversions interrupt postzygotic recombination or survival of recombinants. This work, therefore, has practical implications for breeding programs. Blue catfish is of particular interest with its superior traits of disease resistance against ESC bacterial disease, greater processing yields, and better harvestability [49]. Introgression of superior production and performance traits from the blue catfish genome by interspecific hybridization, followed by one or two generations of backcrossing to achieve homologous chromosome pairs of chromosomes 6, 11, and 24, is a logical step for breeding.

## Methods

### Production of gynogenetic doubled haploid blue and channel catfish

The homozygous catfish used as genome sequencing templates were produced through gynogenesis. The channel catfish, “Coco”, was the same individual used to produce the first channel catfish genome assembly [13] and was produced using established methods [12]. The blue catfish, “Billie”, was produced using a similar approach with the difference of blue catfish eggs fertilized with irradiated channel catfish sperm, and the pressure shock was applied 90 min post-fertilization. Homozygosity was validated by using microsatellite markers [50].

### DNA isolation and sequencing

Genomic DNA was isolated from peripheral red blood cells using standard method of Proteinase K-SDS digestion, ammonium acetate protein precipitation, and precipitation of nucleic acids by 2-propanol. High molecular weight (HMW) DNA was randomly sheared to produce a 350-bp insert library, and paired-end sequences were produced on an Illumina NextSeq 500 platform. For the blue catfish long reads, HMW DNA was sheared with a Covaris® G-tube targeting > 20 kb fragments. Sheared DNA was prepared for PacBio sequencing (Pacific Biosciences, Menlo Park CA) using the SMRTbell™ Template Prep Kit, and size selected with the Blue Pippin (Sage Sciences). Sequencing was performed on a PacBio® RS II System on SMRT®Cell 8Pac V3 cells using P6-C4 chemistry. To target Continuous Long Reads, the libraries were sequenced using 6-h movies on 90 SMRT®Cells. For the channel catfish long reads, HMW libraries were produced as described for blue catfish above and sequencing was performed on a PacBio® Sequel System on 12 LR SMRT®Cells 1 M v3 using SMRTLink version 6 software. Continuous Long Reads were produced using 15-h movies. This channel catfish sample was also run on an 8 M SMRT®Cell on a PacBio® Sequel II System using v7.0 software.

### Sequence assembly

A total of 6,935,942 CLR reads (76,971,401,043 bp) were produced from the blue catfish genome, with a N50 read length of 16,065 bp. A total of 3,696,288 CLR reads (63,729,299,415 bp) were produced from the channel catfish genome with an N50 read length of 25,713 bp. The CLR reads were assembled using Canu v1.8 [51], and sequence accuracy of assembled contigs was improved with two iterations of arrow using the CLR reads followed by one iteration of Freebayes using 77X (blue) or 48X (channel) coverage of Illumina reads [52].

### Optical mapping, hybrid assembly

Bionano optical mapping was performed with blood cells using the Bionano Protocol. Briefly, nucleated blood cells were embedded in agarose and ultra-high molecular weight DNA was isolated according to the Bionano Prep Frozen Blood Protocol (Bionano Genomics, San Diego, CA). A total of 750 ng of DNA was labeled with the Direct Label and Stain (DLS) DNA Labeling kit (Bionano Genomics). Once labeled and stained, the DNA was imaged on the Bionano Saphyr instrument (Bionano Genomics). Images of individual molecules were digitized and assembled into chromosome maps. Super-Scaffolds were produced by Bionano software that incorporated the corrected sequence contigs into the chromosome maps.

### Genetic linkage mapping

The channel catfish genetic linkage map was constructed using single-nucleotide polymorphic markers (SNPs) with 576 fish from three resource families of 192 fish each [14]. The interspecific hybrid linkage map was constructed using SNP markers in 288 backcross progenies with 96 individuals from each of the three backcross families [15]. The blue catfish linkage map was newly constructed for this project using SNP markers of the catfish 690 K SNP array [16]. A total of 141 individuals from three full-sib families of blue catfish were genotyped. The mapping procedures followed the protocols as described [16] with some modifications. Genotype calling of generated signal intensity data in CEL file was performed using the Axiom Analysis Suite software. SNPs classified as “PolyHighResolution” and “NoMinorHom” were remained for further analysis. SNPs with call rate lower than 95%, minor allele frequency (MAF) less than 0.05, or missing value more than 10 were excluded by using SVS software package (SNP & Variation Suite, Version 8.3). The filtered genotyping data were then imported into PLINK 1.0 [53] to examine pedigree information based on pairwise identity-by-state (IBS) distance analysis. Outlier samples were removed once they were detected with significantly larger distances compared with the normal level. Mendelian segregation of SNP markers in the three mapping families were checked using chi-square test of R package “Onemap” [54]. Markers with significant segregation distortion (*p* < 0.001) were eliminated from linkage analysis. Linkage map was constructed using Lep-MAP3 [55]. First, SNP genotyping data from the three families were combined and converted to genotype likelihoods (posteriors) using “linkage2post.awk” script. Then, the “SeparateChromosomes2” module was applied to cluster markers into linkage groups (LGs). The threshold of logarithm of the odds (LOD) score limit of 12 and minimum LG size of 60 (lodLimit = 12 sizeLimit = 60) were applied to form 29 LGs. Singular markers were added to the established LGs by using the “JoinSingles” module with LOD score limit of 5 and minimum difference of 2 (LodLimit = 5 lodDifference = 2). Finally, the module “OrderMarkers2” was used to order makers in each linkage group (LG), which was determined by allowing different recombination probabilities in both sexes. Two rounds of marker ordering procedures were carried out to obtain the order with best likelihood with 10 interactions per each round. After the second round of ordering, genetic distance was calculated with the Kosambi mapping function accounting for both male and female meiosis. Sex-specific recombination rates were then calculated with the same marker order. All genetic linkage maps were drawn with MapChart (version 2.3).

### Assessment and validation of the sequence assembly

The accuracy of the sequence assembly was assessed using MUMmer [56] to compare SNP marker positions on the genetic map with their positions on the genomic sequence scaffolds.

### Genome annotation

The repetitive elements were identified using RepeatModeler 1.0.8 containing RECON [57] and RepeatScout [58] with default parameters. The derived repetitive sequences were searched against Dfam and Repbase [59, 60]. If the sequences were classified as “Unknown”, they were further searched against the non-redundant nucleotide database using blastn 2.11.0 + analysis of repetitive elements. The results, along with a custom library from RepeatMasker, were merged. We used the comprehensive species-specific repeat element library to mask the repeats from known families (replaced with N) and their location information was collected as intergenic. All repetitive regions were soft-masked before annotation of protein-coding genes.

Structural annotation was conducted by three strategies consisting of ab initio, homology, and RNA-seq-based prediction. To conduct ab initio gene prediction, the genome data and RNA-seq short reads (SRR11951631, SRR11951633, SRR11951635, SRR11951637, SRR11951639, SRR11951641, SRR11951643, and SRR392744) were input to the BRAKER2 pipeline [61], which performed iterative gene prediction to train and refine gene models by invoking GeneMark-ES [62] and Augustus [63]. RNA-seq reads were assembled in the genome-guided way by the HISAT2 (v2.1.0) [64] and StringTie (v2.1.4) [65]. Afterward, the genome-guided transcript sets were sent to TransDecoder (https://github.com/TransDecoder) to identify coding sequences by open reading frame (ORF) prediction and homology searches. For homology-based protein prediction, protein sequences of closely related fish species were downloaded from Ensembl, including *Astyanax mexicanus*, *Danio rerio*, *Ictalurus punctatus*, *Oryzias atipes*, and *Pangasianodon hypophthalmus*. Finally, we produced an integrated gene set from MAKER pipeline [66] using the abovementioned three annotations as input datasets. Functional annotation was performed using Diamond (v2.0.15) [67] by alignment of the all assembled unigenes against databases including NR, Swiss-Prot, KEGG, GO, KOG, and eggNOG database. In addition, tRNAscan-SE (v2.1.0) [68] was used to identify tRNA genes with the default settings. The miRNAs, SnRNAs, SnoRNAs, and rRNAs were annotated by searching the Rfam database (http://rfam.xfam.org/) using Infernal (v2.1.0) [69].

### Gene family cluster and phylogenetic analysis

Annotated protein sets of 14 representative species including five catfish species, eight other ray-finned fish, and the lamprey *Petromyzon marinus* were retrieved from NCBI and Ensembl databases. The longest protein isoform was selected to represent each gene. The 3640 Actinopterygii (ray-finned fish) BUSCO genes [70] were used as a benchmark to assess completeness of each gene sets. To identify orthogroups and orthologs from the datasets, we followed the OrthoFinder (v2.5.4) [71] pipeline by invoking DIAMOND and OrthoMCL to call orthogroups based on sequence identity. Finally, the gene set was clustered into 21,019 gene families, and a total of 2977 single-copy gene among these species were identified.

To reveal phylogenetic relationships among channel catfish, blue catfish, and other fish species, the protein sequences of each single-copy orthologous group from the orthology analysis were aligned using MUSCLE [72]. The alignments were then concatenated into a super-gene alignment after trimming by trimAL [73]. From the concatenated datasets, we inferred a Maximum Likelihood (ML) phylogeny tree by RAxML-NG [74] with 1000 bootstrap replicates with TIM2 + I + G4 model using ModelTest-NG [75]. To estimate divergence times, the MCMCtree program in PAML [76] was used for approximate likelihood calculations, based on known approximate divergence times of *L. oculatus* – Neopterygii (298.8–342.5 Ma), *T. rubripes* – *G. aculeatus* (82.0–173.9 Ma), *O. latipes* – *O. niloticus* (83.0–103.8 Ma), *D. rerio* – *A. mexicanus* (132.0–170.0 Ma), *A. melas* – *I. punctatus* (32.4–58.4 Ma), and a time for the root (493.8–652.0 Ma) (http://www.timetree.org/). Additionally, we used CAFÉ [77] to detect gene family expansion and contraction, based on the orthogroups identified by OrthoFinder and the phylogenetic tree with divergence time constructed by MCMCtree.

### Analysis of catfish-specific genes

Genome sequences for *Silurus meridionalis*, *Hemibagrus wyckioides*, *Tachysurus fulvidraco*, *Pangasianodon hypophthalmus*, and *Ameiurus melas* were downloaded from NCBI, and comparatively analyzed against genome sequences of channel catfish and blue catfish. Based on the orthogroups inferred by OrthoFinder, the channel catfish- and blue catfish-specific genes were identified in comparison with other species in the catfish lineage, and the specific genes were identified among one another. A common set of genes shared by all seven catfish was first obtained. The remaining genes specifically present in one or more catfish species were determined. Functional enrichment of these involved genes was then performed using ClusterProfiler 4.0 [78] with the genes of *I. punctatus* or *I. furcatus* assigned as a reference set.

### Whole-genome collinearity analysis

Genome data of channel catfish and blue catfish, as well as those used in the paper such as those for various catfish species were obtained from NCBI. Pairwise collinear analysis was performed by MCScanX (http://chibba.pgml.uga.edu/mcscan2/) with the parameters “-s 4 -m 20 -e 1e-10 -b 2” and visualized by jcvi (https://github.com/tanghaibao/jcvi/wiki/MCscan-(Python-version). The collinearity was visualized with Circos (v0.69.6) [79]. To reveal genome structural rearrangements among species in Siluriformes, pairwise collinear analysis was performed by JCVI with 12,771 single-copy genes, which were identified by Orthofinder among seven catfish species.

### Identification of structural variations of the genome

To identify structural variations in the genomes, MUMmer4 [80] was used to perform whole-genome alignments of genome assemblies of blue catfish (Billie1.0) and channel catfish (Coco2.0) designated as the reference genome. The alignments were filtered with the delta-filter tool and used as input for SyRI [81], which was run with default parameters. SyRI identified syntenic regions between each pair of chromosomes, by which structurally rearranged (non-collinear) regions were simultaneously found. The syntenic regions and structural rearrangements for the genomes were visualized with plotsr (v0.5.3) [82]. Chromosomes 6, 11, and 24 of the blue catfish assembly (Billie1.0) were reverse complemented using seqkit before alignment [83].

### Comparison of recombination frequencies

Four genetic maps were used, including male and female genetic maps of blue catfish (current research), male channel catfish genetic map [14], and male hybrid catfish genetic map constructed from channel catfish × blue catfish F2 backcross families [15]. For all genetic maps, marker orders were compared to physical positions in the channel catfish or blue catfish genome sequence assemblies. The relationship between genetic and physical positions was demonstrated by a scatter plot with the markers’ genetic positions (cM) versus physical positions (Mb). The local recombination rates were estimated and displayed by a smooth line chart in non-overlapping 2 Mb windows with the Loess (locally weighted scatterplot smoothing) method.

### Gene space completeness

The final assembly of channel catfish and blue catfish genomes was assessed using Benchmarking Universal Single-Copy Orthologs (BUSCO) [84] with the lineage database Actinopterygii_odb10. Genome assemblies for the 16 species under comparison were downloaded from NCBI. The 3640 Actinopterygii (ray-finned fish) BUSCO genes were used as a benchmark to assess the genome completeness. Homologous gene pairs between channel catfish and blue catfish were constructed through reciprocal best hit (RBH) method using all-against-all BLASTP (v2.10.1 +).

### Analysis of centromeres

The Xba elements were arranged in head-to-tail tandem arrays. The repeat sequences were extracted from the genome sequences of channel catfish and blue catfish. The positions on each chromosome were located, and their sizes were quantified using the repeat numbers of the Xba elements. Sequences of Xba elements were aligned using Clustal Omega with the online platform of EMBL-EBI (https://www.ebi.ac.uk/Tools/msa/clustalo/). Their tandem nature was confirmed by both sequence analysis and our Southern blot experiments [25]. Similarly, fluorescent in situ hybridization previously conducted in our laboratory demonstrated chromosomal position [26]. The observed copy number difference between channel catfish and blue catfish was confirmed by quantitative PCR.

### Calculation of divergence rates of Xba elements

The average number of substitutions per site (*K*) for each Xba repeat unit was subtotaled. The K value was calculated based on the Jukes-Cantor formula: *K* =  − 300/4 × Ln(1 − *D* × 4/300), the *D* represents the proportion of each Xba repeat unit differing from the consensus sequences [85].

### Quantitative real-time PCR

A quantitative real-time PCR assay was designed and optimized to confirm the relative copy number of Xba elements in the blue and channel catfish genomes. Triplicate reactions were performed in 20 µL with the SsoAdvanced Universal SYBR Green Mix (Bio-Rad Laboratories, Hercules, CA) and 500 nM of each primer (Xba_76F: GTGCTCTTTAKVCGCTCAAAACGC, Xba_145R: AAAAACCACTTTCCTTTGCTCCT) or a single-copy locus on chromosome 12 (Chr12_03F: TCTACAGTTTGGTCCGTATGATC and Chr12_03R: CAATGTCCAGAGAGCTGGCATG) was tested with a temperature gradient, melt curve analysis, and standard curve. The loci were amplified by heating for 3 min at 98 °C, 40 cycles of 10 s at 98 °C and 30 s at 62 °C, followed by a melt curve on a CFX96 Touch Real-Time PCR Detection System (Bio-Rad Laboratories). Normalized quantities were calculated from three replicates each from four channel catfish and four blue catfish using the 2^−ΔΔCt^ method [86].

## Supplementary Information

**Additional file 1: Figure S1.** Comparison of sequences included in the 29 chromosomes of channel catfish genome sequence assembly Coco_2.0 (blue) and Coco_1.2 (red). **Figure S2.** Xba element sequences are more conserved within channel catfish than in blue catfish. Xba repeats in blue catfish are more divergent even within a single centromere. Shown here are sequence variations of Xba elements from chromosome 2 of blue catfish. **Figure S3.** Genome level comparison of Billie_1.0 with AU_DB_2.0. Sequence of Wang et al. are on the Y-axis, plotted against Billie_1.0 on the X-axis. Note the potential mis-assemblies by Wang et al., with major artifactual inversions in their assembly indicated within blue oval circles. **Figure S4.** Chromosomal inversions not reported (chromosome 6, 11, and 24) or created as an assembly artifact (Chromosome 7) from Wang et al.27. In each of the four chromosomes, the upper panels present dot plot alignments of our assembly Billie_1.0 (X-axis) with the assembly of Wang et al. Blue lines represent forward alignment and red lines represent reverse alignment. The lower panels display the dis-concordance of the assembly of Wang et al. with the genetic linkage map of blue catfish. **Supplementary Table S1.** Summary of PacBio contiguous long read (CLR) sequencing and Illumina sequencing. **Supplementary Table S2.** Optical map and hybrid assembly. **Supplementary Table S3.** Comparison of the assemblies of channel catfish and blue catfish reference genomes. **Supplementary Table S4.** Summary of structural variations between channel catfish genome and blue catfish genome. **Supplementary Table S5.** Summary of structural variations (SV) greater than 1Mb between channel catfish genome and blue catfish genome. **Supplementary Table S6.** Gene contents in the inversional segments. **Supplementary Table S8.** Numbers of genes on each of the 29 chromosomes of channel catfish and blue catfish. **Supplementary Table S9.** Comparison of annotated protein coding genes from selected teleost species. Channel catfish and blue catfish are highlighted. **Supplementary Table S13.** Categories of repetitive elements in the blue catfish (*Ictalurus furcatus*) genome. **Supplementary Table S14.** Categories of repetitive elements in the channel catfish (*Ictalurus punctatus*) genome. **Supplementary Table S15.** Centromere positions in Blue and Channel Catfish chromosomes measured as span of Xba elements. **Supplementary Table S16.** Centromeric and telomeric sequences for channel and blue catfish chromosome assemblies. Position numbers in red are highlighted as different between channel and blue. **Supplementary Table S17.** Primers and expected amplicons of junction PCR.

**Additional file 2: Supplementary Table S7.** Shared markers between blue catfish and channel catfish. **Supplementary Table S10.** Gene annotation of 508 gene families that were specific to channel catfish and blue catfish compared with other 14 representative fish species. **Supplementary Table S11.** Annotation of 1127 genes within 732 families that are specific to channel catfish. **Supplementary Table S12.** Annotation of 606 genes within 434 families that are specific to blue catfish.

## Data Availability

All data generated or analyzed during this study are included in this published article, its supplementary information files and publicly available repositories. The assembled sequence data have been submitted to the BioProject database under accessions PRJNA327588 for channel catfish and PRJNA834118 for blue catfish. The Coco_2.0 and Billie_1.0 whole-genome assemblies have been deposited at DDBJ/EMBL/GenBank under the accessions LBML02000000 and JAMCCP000000000, respectively. The raw PacBio and Illumina reads are now available in GenBank. Instead of a separate SRA accession number, NCBI points to the BioProject to get to the SRA data. The citation for the blue catfish is PRJNA834118 and for channel is PRJNA281269.

## References

[1] Catalog of fishes: Species by family/subfamily. http://research.calacademy.org/research/ichthyology/catalog/SpeciesByFamily.asp. Accessed 6 Apr 2022.

[2] Sullivan JP, Lundberg JG, Hardman M (2006). A phylogenetic analysis of the major groups of catfishes (Teleostei: Siluriformes) using rag1 and rag2 nuclear gene sequences. Mol Phylogenet Evol.

[3] The State of World Fisheries and Aquaculture. 2020. https://www.fao.org/documents/card/en/c/ca9229en/. Accessed 6 Apr 2022.

[4] Dunham RA, Elaswad A (2018). Catfish biology and farming. Annu Rev Anim Biosci.

[5] Dunham R, Masser M. Production of hybrid catfish. In: Southern Regional Aquaculture Center. Publication 190. Stoneville; 1998.

[6] Geng X, Sha J, Liu S, Bao L, Zhang J, Wang R, Yao J, Li C, Feng J, Sun F (2015). A genome-wide association study in catfish reveals the presence of functional hubs of related genes within QTLs for columnaris disease resistance. BMC Genomics.

[7] Zhou T, Liu S, Geng X, Jin Y, Jiang C, Bao L, Yao J, Zhang Y, Zhang J, Sun L (2017). GWAS analysis of QTL for enteric septicemia of catfish and their involved genes suggest evolutionary conservation of a molecular mechanism of disease resistance. Mol Genet Genomics.

[8] Ayala FJ, Coluzzi M (2005). Chromosome speciation: humans, Drosophila, and mosquitoes. Proc Natl Acad Sci U S A.

[9] Fuller ZL, Leonard CJ, Young RE, Schaeffer SW, Phadnis N (2018). Ancestral polymorphisms explain the role of chromosomal inversions in speciation. PLoS Genet.

[10] Noor MA, Grams KL, Bertucci LA, Reiland J (2001). Chromosomal inversions and the reproductive isolation of species. Proc Natl Acad Sci U S A.

[11] Coyne JO, Orr HA (2004). Speciation.

[12] Waldbieser GC, Bosworth BG, Quiniou SM. Production of viable homozygous, doubled haploid channel catfish (*Ictalurus punctatus*). Mar Biotechnol (NY). 2010;12:380–5. 10.1007/s10126-009-9221-2 19707826

[13] Liu Z, Liu S, Yao J, Bao L, Zhang J, Li Y, Jiang C, Sun L, Wang R, Zhang Y (2016). The channel catfish genome sequence provides insights into the evolution of scale formation in teleosts. Nat Commun.

[14] Li Y, Liu S, Qin Z, Waldbieser G, Wang R, Sun L, Bao L, Danzmann RG, Dunham R, Liu Z (2015). Construction of a high-density, high-resolution genetic map and its integration with BAC-based physical map in channel catfish. DNA Res.

[15] Liu S, Li Y, Qin Z, Geng X, Bao L, Kaltenboeck L, Kucuktas H, Dunham R, Liu Z (2016). High-density interspecific genetic linkage mapping provides insights into genomic incompatibility between channel catfish and blue catfish. Anim Genet.

[16] Zeng Q, Fu Q, Li Y, Waldbieser G, Bosworth B, Liu S, Yang Y, Bao L, Yuan Z, Li N, Liu Z (2017). Development of a 690 K SNP array in catfish and its application for genetic mapping and validation of the reference genome sequence. Sci Rep.

[17] Fishman L, Stathos A, Beardsley PM, Williams CF, Hill JP (2013). Chromosomal rearrangements and the genetics of reproductive barriers in mimulus (monkey flowers). Evolution.

[18] Simao FA, Waterhouse RM, Ioannidis P, Kriventseva EV, Zdobnov EM (2015). BUSCO: assessing genome assembly and annotation completeness with single-copy orthologs. Bioinformatics.

[19] Chen W, Zou M, Li Y, Zhu S, Li X, Li J (2021). Sequencing an F1 hybrid of Silurus asotus and S. meridionalis enabled the assembly of high-quality parental genomes. Sci Rep.

[20] Zheng S, Shao F, Tao W, Liu Z, Long J, Wang X, et al. Chromosome-level assembly of southern catfish (*Silurus meridionalis*) provides insights into visual adaptation to nocturnal and benthic lifestyles. Mol Ecol Resour. 2021;21:1575–92. 10.1111/1755-0998.13338 33503304

[21] Shao F, Pan H, Li P, Ni L, Xu Y, Peng Z. Chromosome-level genome assembly of the Asian red-tail catfish (*Hemibagrus wyckioides*). Front Genet. 2021;12:747684. 10.3389/fgene.2021.747684 PMC8546334 34712270

[22] Gong G, Dan C, Xiao S, Guo W, Huang P, Xiong Y, Wu J, He Y, Zhang J, Li X (2018). Chromosomal-level assembly of yellow catfish genome using third-generation DNA sequencing and Hi-C analysis. Gigascience.

[23] Kim OTP, Nguyen PT, Shoguchi E, Hisata K, Vo TTB, Inoue J, et al. A draft genome of the striped catfish, *Pangasianodon hypophthalmus*, for comparative analysis of genes relevant to development and a resource for aquaculture improvement. BMC Genomics. 2018;19:733. 10.1186/s12864-018-5079-x PMC6173838 30290758

[24] Yuan Z, Zhou T, Bao L, Liu S, Shi H, Yang Y, et al. The annotation of repetitive elements in the genome of channel catfish (*Ictalurus punctatus*). PLoS One. 2018;13(5):e01973s71. 10.1371/journal.pone.0197371. 10.1371/journal.pone.0197371 PMC5953449 29763462

[25] Liu Z, Li P, Dunham RA. Characterization of an A/T-rich family of sequences from channel catfish (*Ictalurus punctatus*). Mol Mar Biol Biotechnol. 1998;7:232–9. 9701618

[26] Quiniou SM, Wolters WR, Waldbieser GC. Localization of Xba repetitive elements to channel catfish (*Ictalurus punctatus*) centromeres via fluorescence in situ hybridization. Anim Genet. 2005;36:353–4. 10.1111/j.1365-2052.2005.01304.x 16026349

[27] Wang H, Su B, Butts IAE, Dunham RA, Wang X. Chromosome-level assembly and annotation of the blue catfish *Ictalurus furcatus, *an aquaculture species for hybrid catfish reproduction, epigenetics, and heterosis studies. Gigascience. 2022;11:giac070. 10.1093/gigascience/giac070 PMC9270728 35809049

[28] Matschiner M, Barth JMI, Tørresen OK, Star B, Baalsrud HT, Brieuc MSO, Pampoulie C, Bradbury I, Jakobsen KS, Jentoft S (2022). Supergene origin and maintenance in Atlantic cod. Nat Ecol Evol.

[29] Tan S, Zhou T, Wang W, Jin Y, Wang X, Geng X, Luo J, Yuan Z, Yang Y, Shi H (2018). GWAS analysis using interspecific backcross progenies reveals superior blue catfish alleles responsible for strong resistance against enteric septicemia of catfish. Mol Genet Genomics.

[30] Rhie A, McCarthy SA, Fedrigo O, Damas J, Formenti G, Koren S, Uliano-Silva M, Chow W, Fungtammasan A, Kim J (2021). Towards complete and error-free genome assemblies of all vertebrate species. Nature.

[31] Howe K, Clark MD, Torroja CF, Torrance J, Berthelot C, Muffato M, Collins JE, Humphray S, McLaren K, Matthews L (2013). The zebrafish reference genome sequence and its relationship to the human genome. Nature.

[32] McGaugh SE, Gross JB, Aken B, Blin M, Borowsky R, Chalopin D, Hinaux H, Jeffery WR, Keene A, Ma L (2014). The cavefish genome reveals candidate genes for eye loss. Nat Commun.

[33] Lien S, Koop BF, Sandve SR, Miller JR, Kent MP, Nome T, Hvidsten TR, Leong JS, Minkley DR, Zimin A (2016). The Atlantic salmon genome provides insights into rediploidization. Nature.

[34] Du K, Stock M, Kneitz S, Klopp C, Woltering JM, Adolfi MC, Feron R, Prokopov D, Makunin A, Kichigin I (2020). The sterlet sturgeon genome sequence and the mechanisms of segmental rediploidization. Nat Ecol Evol.

[35] Lin X, Huang Y, Jiang D, Chen H, Deng S, Zhang Y, et al. Chromosomal-level genome assembly of silver sillago (*Sillago sihama*). Genome Biol Evol. 2021;13:evaa272. 10.1093/gbe/evaa272 PMC7875006 33367716

[36] Chen S, Zhang G, Shao C, Huang Q, Liu G, Zhang P, Song W, An N, Chalopin D, Volff JN (2014). Whole-genome sequence of a flatfish provides insights into ZW sex chromosome evolution and adaptation to a benthic lifestyle. Nat Genet.

[37] Xu P, Xu J, Liu G, Chen L, Zhou Z, Peng W, Jiang Y, Zhao Z, Jia Z, Sun Y (2019). The allotetraploid origin and asymmetrical genome evolution of the common carp Cyprinus carpio. Nat Commun.

[38] Brawand D, Wagner CE, Li YI, Malinsky M, Keller I, Fan S, Simakov O, Ng AY, Lim ZW, Bezault E (2014). The genomic substrate for adaptive radiation in African cichlid fish. Nature.

[39] Tine M, Kuhl H, Gagnaire PA, Louro B, Desmarais E, Martins RS, Hecht J, Knaust F, Belkhir K, Klages S (2014). European sea bass genome and its variation provide insights into adaptation to euryhalinity and speciation. Nat Commun.

[40] Shen EZ, Chen H, Ozturk AR, Tu S, Shirayama M, Tang W, Ding YH, Dai SY, Weng Z, Mello CC (2018). Identification of piRNA binding sites reveals the argonaute regulatory landscape of the C. elegans germline. Cell.

[41] Aravin AA, Sachidanandam R, Girard A, Fejes-Toth K, Hannon GJ (2007). Developmentally regulated piRNA clusters implicate MILI in transposon control. Science.

[42] Batista PJ, Ruby JG, Claycomb JM, Chiang R, Fahlgren N, Kasschau KD, et al. PRG-1 and 21U-RNAs interact to form the piRNA complex required for fertility in *C. elegans*. Mol Cell. 2008;31:67–78. 10.1016/j.molcel.2008.06.002 PMC2570341 18571452

[43] Siomi MC, Sato K, Pezic D, Aravin AA (2011). PIWI-interacting small RNAs: the vanguard of genome defence. Nat Rev Mol Cell Biol.

[44] Bengten E, Clem LW, Miller NW, Warr GW, Wilson M (2006). Channel catfish immunoglobulins: repertoire and expression. Dev Comp Immunol.

[45] Arce HM, Lundberg JG, O'Leary MA (2017). Phylogeny of the North American catfish family Ictaluridae (Teleostei: Siluriformes) combining morphology, genes and fossils. Cladistics.

[46] Roberts TR (1983). Revision of the South and Southeast Asian Sisorid catfish genus Bagarius, with description of a new species from the Mekong. Copeia.

[47] Melters DP, Bradnam KR, Young HA, Telis N, May MR, Ruby JG, Sebra R, Peluso P, Eid J, Rank D (2013). Comparative analysis of tandem repeats from hundreds of species reveals unique insights into centromere evolution. Genome Biol.

[48] Ichikawa K, Tomioka S, Suzuki Y, Nakamura R, Doi K, Yoshimura J, Kumagai M, Inoue Y, Uchida Y, Irie N (1833). Centromere evolution and CpG methylation during vertebrate speciation. Nat Commun.

[49] Abdelrahman H, ElHady M, Alcivar-Warren A, Allen S, Al-Tobasei R, Bao L, Beck B, Blackburn H, Bosworth B, Buchanan J (2017). Aquaculture genomics, genetics and breeding in the United States: current status, challenges, and priorities for future research. BMC Genomics.

[50] Waldbieser GC, Bosworth BG. A standardized microsatellite marker panel for parentage and kinship analyses in channel catfish *Ictalurus punctatus*. Anim Genet. 2013;44:476–9. 10.1111/age.12017 23216371

[51] Koren S, Walenz BP, Berlin K, Miller JR, Bergman NH, Phillippy AM (2017). Canu: scalable and accurate long-read assembly via adaptive k-mer weighting and repeat separation. Genome Res.

[52] Garrison E, Marth G. Haplotype-based variant detection from short-read sequencing. arXiv:12073907v2[q-bioGN). 2012.

[53] Purcell S, Neale B, Todd-Brown K, Thomas L, Ferreira MA, Bender D, Maller J, Sklar P, de Bakker PI, Daly MJ, Sham PC (2007). PLINK: a tool set for whole-genome association and population-based linkage analyses. Am J Hum Genet.

[54] Margarido GR, Souza AP, Garcia AA (2007). OneMap: software for genetic mapping in outcrossing species. Hereditas.

[55] Rastas P (2017). Lep-MAP3: robust linkage mapping even for low-coverage whole genome sequencing data. Bioinformatics.

[56] Kurtz S, Phillippy A, Delcher AL, Smoot M, Shumway M, Antonescu C, Salzberg SL (2004). Versatile and open software for comparing large genomes. Genome Biol.

[57] Bao Z, Eddy SR (2002). Automated de novo identification of repeat sequence families in sequenced genomes. Genome Res.

[58] Price AL, Jones NC, Pevzner PA (2005). De novo identification of repeat families in large genomes. Bioinformatics.

[59] Hubley R, Finn RD, Clements J, Eddy SR, Jones TA, Bao W, Smit AF, Wheeler TJ (2016). The Dfam database of repetitive DNA families. Nucleic Acids Res.

[60] Bao W, Kojima KK, Kohany O (2015). Repbase Update, a database of repetitive elements in eukaryotic genomes. Mob DNA.

[61] Bruna T, Hoff KJ, Lomsadze A, Stanke M, Borodovsky M (2021). BRAKER2: automatic eukaryotic genome annotation with GeneMark-EP+ and AUGUSTUS supported by a protein database. NAR Genom Bioinform.

[62] Lomsadze A, Ter-Hovhannisyan V, Chernoff YO, Borodovsky M (2005). Gene identification in novel eukaryotic genomes by self-training algorithm. Nucleic Acids Res.

[63] Stanke M, Keller O, Gunduz I, Hayes A, Waack S, Morgenstern B (2006). AUGUSTUS: ab initio prediction of alternative transcripts. Nucleic Acids Res.

[64] Kim D, Paggi JM, Park C, Bennett C, Salzberg SL (2019). Graph-based genome alignment and genotyping with HISAT2 and HISAT-genotype. Nat Biotechnol.

[65] Pertea M, Pertea GM, Antonescu CM, Chang TC, Mendell JT, Salzberg SL (2015). StringTie enables improved reconstruction of a transcriptome from RNA-seq reads. Nat Biotechnol.

[66] Campbell MS, Holt C, Moore B, Yandell M (2014). Genome annotation and curation using MAKER and MAKER-P. Curr Protoc Bioinformatics.

[67] Buchfink B, Xie C, Huson DH (2015). Fast and sensitive protein alignment using DIAMOND. Nat Methods.

[68] Chan PP, Lin BY, Mak AJ, Lowe TM (2021). tRNAscan-SE 2.0: improved detection and functional classification of transfer RNA genes. Nucleic Acids Res.

[69] Nawrocki EP, Kolbe DL, Eddy SR (2009). Infernal 1.0: inference of RNA alignments. Bioinformatics.

[70] Waterhouse RM, Seppey M, Simao FA, Manni M, Ioannidis P, Klioutchnikov G, Kriventseva EV, Zdobnov EM (2018). BUSCO applications from quality assessments to gene prediction and phylogenomics. Mol Biol Evol.

[71] Emms DM, Kelly S (2019). OrthoFinder: phylogenetic orthology inference for comparative genomics. Genome Biol.

[72] Edgar RC. High-accuracy alignment ensembles enable unbiased assessments of sequence homology and phylogeny. bioRxiv. 2022 10.1101/2021.06.20.449169. 10.1038/s41467-022-34630-w PMC9664440 36379955

[73] Capella-Gutierrez S, Silla-Martinez JM, Gabaldon T (2009). trimAl: a tool for automated alignment trimming in large-scale phylogenetic analyses. Bioinformatics.

[74] Kozlov AM, Darriba D, Flouri T, Morel B, Stamatakis A (2019). RAxML-NG: a fast, scalable and user-friendly tool for maximum likelihood phylogenetic inference. Bioinformatics.

[75] Darriba D, Posada D, Kozlov AM, Stamatakis A, Morel B, Flouri T (2020). ModelTest-NG: a new and scalable tool for the selection of DNA and protein evolutionary models. Mol Biol Evol.

[76] Yang Z (2007). PAML 4: phylogenetic analysis by maximum likelihood. Mol Biol Evol.

[77] Mendes FK, Vanderpool D, Fulton B, Hahn MW (2020). CAFE 5 models variation in evolutionary rates among gene families. Bioinformatics.

[78] Wu T, Hu E, Xu S, Chen M, Guo P, Dai Z, Feng T, Zhou L, Tang W, Zhan L (2021). clusterProfiler 4.0: a universal enrichment tool for interpreting omics data. Innovation (Camb).

[79] Krzywinski M, Schein J, Birol I, Connors J, Gascoyne R, Horsman D, Jones SJ, Marra MA (2009). Circos: an information aesthetic for comparative genomics. Genome Res.

[80] Marcais G, Delcher AL, Phillippy AM, Coston R, Salzberg SL, Zimin A (2018). MUMmer4: a fast and versatile genome alignment system. PLoS Comput Biol.

[81] Goel M, Sun H, Jiao WB, Schneeberger K (2019). SyRI: finding genomic rearrangements and local sequence differences from whole-genome assemblies. Genome Biol.

[82] Goel M, Schneeberger K (2022). plotsr: visualizing structural similarities and rearrangements between multiple genomes. Bioinformatics.

[83] Shen W, Le S, Li Y, Hu F (2016). SeqKit: a cross-platform and ultrafast toolkit for FASTA/Q file manipulation. PLoS ONE.

[84] Manni M, Berkeley MR, Seppey M, Simao FA, Zdobnov EM (2021). BUSCO update: novel and streamlined workflows along with broader and deeper phylogenetic coverage for scoring of eukaryotic, prokaryotic, and viral genomes. Mol Biol Evol.

[85] Waterston RH, Lindblad-Toh K, Birney E, Rogers J, Abril JF, Agarwal P, Agarwal R, Ainscough R, Alexandersson M, Mouse Genome Sequencing C (2002). Initial sequencing and comparative analysis of the mouse genome. Nature.

[86] Schmittgen TD, Livak KJ (2008). Analyzing real-time PCR data by the comparative C(T) method. Nat Protoc.

