# Supplementary material for: Reference genomes of channel catfish and blue catfish reveal multiple pericentric chromosome inversions
Source: BMC Biol. 2023 Apr 3;21:67. doi: 10.1186/s12915-023-01556-8 (PMC10071708; doi:10.1186/s12915-023-01556-8)
Supplement: Supplementary file 1 — Additional file 1: Figure S1. Comparison of sequences included in the 29 chromosomes of channel catfish genome sequence assembly Coco_2.0 (blue) and Coco_1.2 (red). Figure S2. Xba element sequences are more conserved within channel catfish than in blue catfish. Xba repeats in blue catfish are more divergent even within a single centromere. Shown here are sequence variations of Xba elements from chromosome 2 of blue catfish. Figure S3. Genome level comparison of Billie_1.0 with AU_DB_2.0. Sequence of Wang et al. are on the Y-axis, plotted against Billie_1.0 on the X-axis. Note the potential mis-assemblies by Wang et al., with major artifactual inversions in their assembly indicated within blue oval circles. Figure S4. Chromosomal inversions not reported (chromosome 6, 11, and 24) or created as an assembly artifact (Chromosome 7) from Wang et al.27. In each of the four chromosomes, the upper panels present dot plot alignments of our assembly Billie_1.0 (X-axis) with the assembly of Wang et al. Blue lines represent forward alignment and red lines represent reverse alignment. The lower panels display the dis-concordance of the assembly of Wang et al. with the genetic linkage map of blue catfish. Supplementary Table S1. Summary of PacBio contiguous long read (CLR) sequencing and Illumina sequencing. Supplementary Table S2. Optical map and hybrid assembly. Supplementary Table S3. Comparison of the assemblies of channel catfish and blue catfish reference genomes. Supplementary Table S4. Summary of structural variations between channel catfish genome and blue catfish genome. Supplementary Table S5. Summary of structural variations (SV) greater than 1Mb between channel catfish genome and blue catfish genome. Supplementary Table S6. Gene contents in the inversional segments. Supplementary Table S8. Numbers of genes on each of the 29 chromosomes of channel catfish and blue catfish. Supplementary Table S9. Comparison of annotated protein coding genes from selected teleost speci [file 12915_2023_1556_MOESM1_ESM.docx]

**Supplementary Materials for**

**Reference genomes of sibling species channel catfish and blue catfish reveal multiple pericentric chromosome inversions**

Geoffrey C. Waldbieser et al.

Corresponding author: Zhanjiang Liu, johnliu@syr.edu;


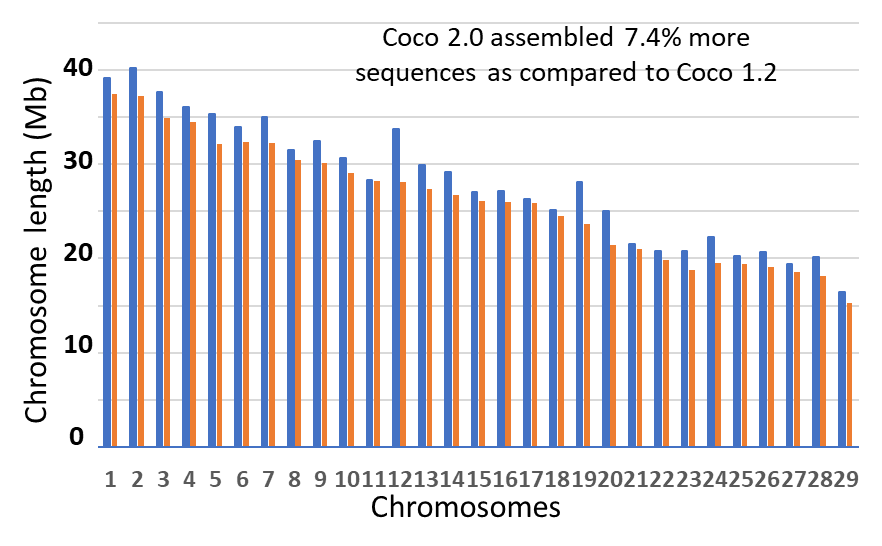


**Figure S1. Comparison of sequences included in the 29 chromosomes of channel catfish genome sequence assembly Coco_2.0 (blue) and Coco_1.2 (red).**

**Figure S2. Xba element sequences are more conserved within channel catfish than in blue catfish.** Xba repeats in blue catfish are more divergent even within a single centromere. Shown here are sequence variations of Xba elements from chromosome 2 of blue catfish.


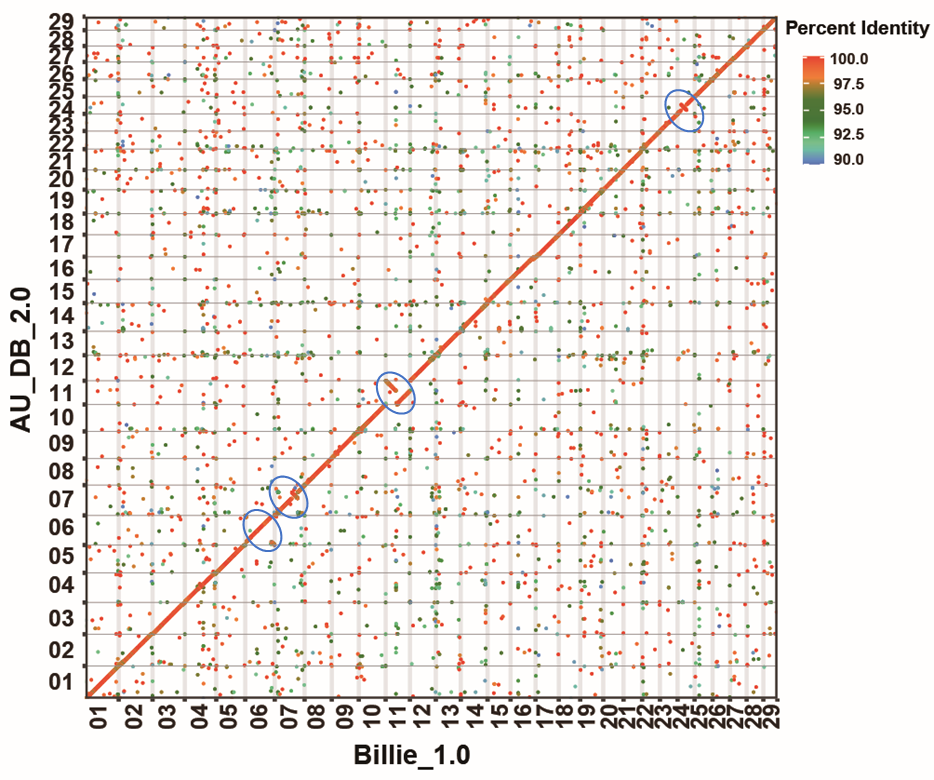


**Figure S3. Genome level comparison of Billie_1.0 with AU_DB_2.0.** Sequence of Wang et al. are on the Y-axis, plotted against Billie_1.0 on the X-axis. Note the potential mis-assemblies by Wang et al., with major artifactual inversions in their assembly indicated within blue oval circles.


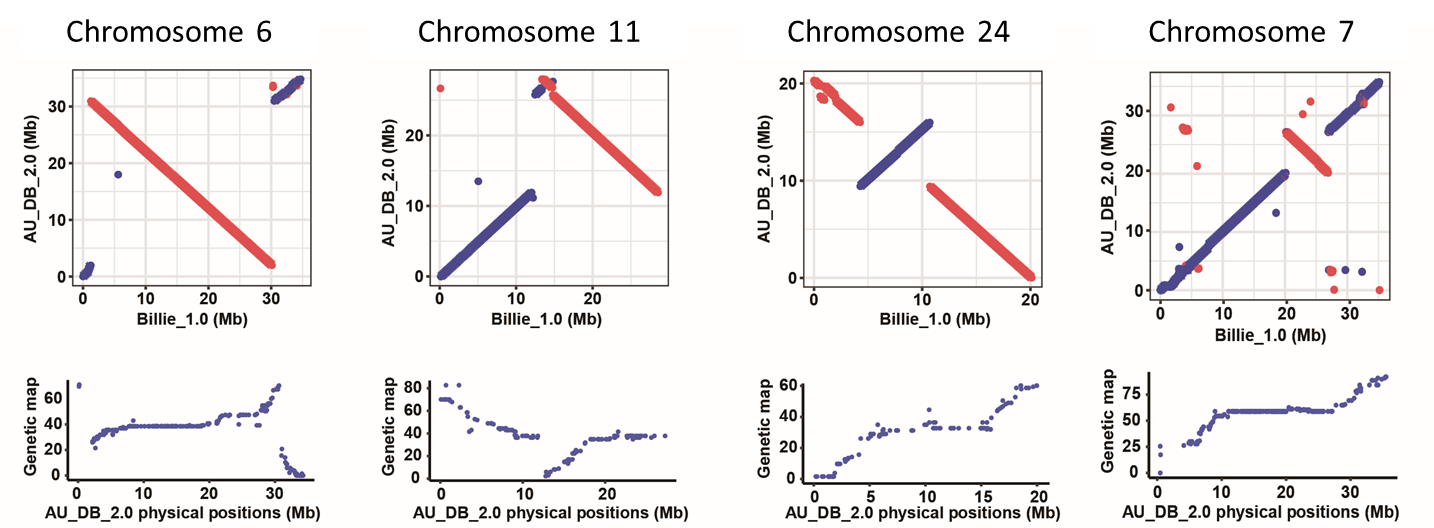


**Figure S4**. **Chromosomal inversions not reported (chromosome 6, 11, and 24) or created as an assembly artifact (Chromosome 7) from Wang et al.^27^.** In each of the four chromosomes, the upper panels present dot plot alignments of our assembly Billie_1.0 (X-axis) with the assembly of Wang et al. Blue lines represent forward alignment and red lines represent reverse alignment. The lower panels display the dis-concordance of the assembly of Wang et al. with the genetic linkage map of blue catfish.

**Supplementary Table S1. Summary of PacBio contiguous long read (CLR) sequencing and Illumina sequencing**

|  | **Blue (Billie)** | **Channel (Coco)** |
| --- | --- | --- |
| **PacBio data** ^1^ |  |  |
| Total length (bp) | 76,971,401,043 | 63,729,299,415 |
| Total reads | 6,935,942 | 3,696,288 |
| N50 length (bp) | 16,065 | 25,713 |
|  |  |  |
| **Canu corrected/trimmed** |  |  |
| Trimmed length (bp) | 30,565,939,086 | 30,688,038,302 |
| Trimmed reads | 1,766,550 | 818,092 |
|  |  |  |
| **Canu primary contigs** |  |  |
| Total length (bp) | 826,206,075 | 846,974,322 |
| Total contigs | 1070 | 665 |
| N50 length (bp) | 6,717,455 | 11,679,570 |
| L50 contigs | 34 | 22 |
|  |  |  |
| **Illumina sequence ^2^** |  |  |
| Total length (bp) | 63,979,027,654 | 40,930,733,140 |
| Total reads | 495,543,520 | 344,821,544 |

^1^ Minimum length 1,000 bp

^2^ Utilized in Freebayes correction of Canu contigs

**Supplementary Table S2. Optical map and hybrid assembly**

|  | **Blue (Billie)** | **Channel (Coco)** |
| --- | --- | --- |
| **Bionano Molecules** | | |
| Molecules | 867,789 | 712,383 |
| Total Bases (Gb) | 263 | 196 |
| Avg Length (kb) | 303 | 275 |
| Molecule N50 (kb) | 300 | 264 |
|  |  |  |
| **Bionano Assembly** | | |
| Bionano Maps | 75 | 86 |
| Genome Map Length (kb) | 860,184 | 840,618 |
| Genome Map N50 (kb) | 23,990 | 19,395 |
| Genome Coverage | 306 X | 233 X |
|  |  |  |
| **Bionano- Sequence + Molecule Scaffolds** | | |
| Number of Scaffolds | 64 | 79 |
| Total Length (bp) | 823,818,031 | 833,834,466 |
| N50 Length (bp) | 25,113,217 | 19,747,932 |
| L50 | 14 | 17 |
|  |  |  |

**Supplementary Table S3**. Comparison of the assemblies of channel catfish and blue catfish reference genomes.

|  | Coco_2.0 | | |  | Billie_1.0 | | |
| --- | --- | --- | --- | --- | --- | --- | --- |
|  | Total Bp | Gaps | Gap Length (%) |  | Total Bp | Gaps | Gap Length (%) |
| Chr01 | 39,107,963 | 2 | 8,661 (0.02) |  | 39,156,680 | 6 | 112,855 (0.3) |
| Chr02 | 40,225,253 | 5 | 150,800 (0.37) |  | 39,819,744 | 7 | 753,427 (1.9) |
| Chr03 | 37,689,791 | 3 | 130,009 (0.34) |  | 38,033,689 | 4 | 31,893 (0.1) |
| Chr04 | 36,068,007 | 5 | 88,047 (0.24) |  | 36,961,748 | 8 | 640,696 (1.7) |
| Chr05 | 35,307,646 | 7 | 377,122 (1.07) |  | 34,185,896 | 9 | 616,641 (1.8) |
| Chr06 | 33,913,446 | 2 | 7,631 (0.02) |  | 34,706,042 | 2 | 2,424 (0.0) |
| Chr07 | 35,057,770 | 2 | 200 (0.00) |  | 35,172,167 | 5 | 744,484 (2.1) |
| Chr08 | 31,480,639 | 2 | 10,150 (0.03) |  | 32,059,992 | 5 | 426,294 (1.3) |
| Chr09 | 32,429,570 | 2 | 57,701 (0.18) |  | 31,799,861 | 6 | 624,685 (2.0) |
| Chr10 | 30,657,734 | 0 | 0 (0.00) |  | 31,605,418 | 8 | 212,429 (0.7) |
| Chr11 | 28,357,196 | 1 | 100 (0.00) |  | 28,650,356 | 2 | 148,263 (0.5) |
| Chr12 | 33,762,910 | 2 | 170,539 (0.51) |  | 30,432,001 | 6 | 129,968 (0.4) |
| Chr13 | 29,879,606 | 0 | 0 (0.00) |  | 29,373,386 | 5 | 437,791 (1.5) |
| Chr14 | 29,208,522 | 2 | 15,280 (0.05) |  | 31,559,510 | 4 | 2,351,282 (7.5) |
| Chr15 | 27,035,493 | 1 | 6,909 (0.03) |  | 27,161,549 | 5 | 111,815 (0.4) |
| Chr16 | 27,127,331 | 1 | 100 (0.00) |  | 29,401,232 | 3 | 4,706 (0.0) |
| Chr17 | 26,294,090 | 3 | 124,978 (0.48) |  | 27,384,689 | 5 | 494,683 (1.8) |
| Chr18 | 25,178,057 | 1 | 100 (0.00) |  | 25,248,234 | 5 | 12,531 (0.5) |
| Chr19 | 28,067,236 | 5 | 1,062,600 (3.79) |  | 27,077,946 | 7 | 176,419 (0.7) |
| Chr20 | 24,995,078 | 1 | 23,450 (0.09) |  | 22,829,125 | 4 | 289,598 (1.2) |
| Chr21 | 21,541,656 | 2 | 130,340 (0.61) |  | 21,900,473 | 3 | 285,728 (1.3) |
| Chr22 | 20,758,072 | 2 | 200 (0.00) |  | 21,039,390 | 6 | 709,973 (3.4) |
| Chr23 | 20,789,747 | 2 | 200 (0.00) |  | 20,842,748 | 1 | 197,114 (0.9) |
| Chr24 | 22,224,706 | 1 | 195,782 (0.88) |  | 20,112,729 | 2 | 1,627 (0.0) |
| Chr25 | 20,218,528 | 1 | 100 (0.00) |  | 21,152,511 | 2 | 94,811 (0.4) |
| Chr26 | 20,701,421 | 2 | 27,152 (0.13) |  | 20,560,872 | 3 | 147,966 (0.7) |
| Chr27 | 19,431,687 | 0 | 0 (0.00) |  | 19,554,630 | 3 | 230,697 (1.2) |
| Chr28 | 20,119,889 | 5 | 307,884 (1.53) |  | 19,738,181 | 6 | 1,046,990 (5.3) |
| Chr29 | 16,467,644 | 5 | 225,621 (1.37) |  | 16,596,099 | 7 | 600,692 (3.6) |
| **SUM** | **814,096,688** | **67** | **3,121,656 (0.38)** |  | **815,116,898** | **139** | **11,638,482 (1.4)** |

**Supplementary Table S4**. **Summary of structural variations between channel catfish genome and blue catfish genome**

| SV type | SV type abbr | Total counts |
| --- | --- | --- |
| Insertion | INS | 2,435 |
| Deletion | DEL | 1,838 |
| Copy gain | CPG | 466 |
| Copy loss | CPL | 2,322 |
| Tandem repeat | TDM | 1,348 |
| Duplication | DUP, INVDP | 17,592 |
| Inversion | INV | 179 |
| Translocation | TRANS, INVTR | 3,413 |
| Total | - | 29,593 |

**Supplementary Table S5. Summary of structural variations (SV) greater than 1Mb between channel catfish genome and blue catfish genome**

| **Chr** | **Length of SV in Coco_2.0** | **Start**  **in Coco_2.0 (bp)** | **End**  **in Coco_2.0 (bp)** | **Length of SV in Billie_1.0** | **Start**  **in Billie_1.0 (bp)** | **End**  **in Billie_1.0 (bp)** | **Reverse start in Billie_1.0 (bp)** | **Reverse end in Billie_1.0 (bp)** |
| --- | --- | --- | --- | --- | --- | --- | --- | --- |
| chr06 | 29,661,124 | 3,815,287 | 33,476,410 | 29,808,446 | 468,710 | 30,277,155 | 4,428,888 | 34,237,333 |
| chr11 | 16,699,138 | 218,608 | 16,917,745 | 16,999,704 | 11,638,546 | 28,638,249 | 12,108 | 17,011,811 |
| chr16 | 2,147,156 | 24,067,596 | 26,214,751 | 4,195,894 | 24,332,357 | 28,528,250 | - | - |
| chr24 | 15,968,512 | 80,759 | 16,049,270 | 14,248,326 | 5,861,680 | 20,110,005 | 12,108 | 17,011,811 |

**Supplementary Table S6. Gene contents in the inversional segments**

|  | Chr | Start(bp) | End(bp) | Number of genes | Orthogroups | | single-copy gene | | Specific  gene | |
| --- | --- | --- | --- | --- | --- | --- | --- | --- | --- | --- |
| *I. punctatus* | 6 | 3,815,287 | 33,476,410 | 728 | 690 | 416 | | 38 | |  |
| *I. furcatus* | 6 | 468,709 | 30,277,154 | 719 | 681 | 416 | | 38 | |  |
| *I. punctatus* | 11 | 218,608 | 16,917,745 | 416 | 394 | 224 | | 22 | |  |
| *I. furcatus* | 11 | 11,638,545 | 28,638,248 | 416 | 398 | 224 | | 18 | |  |
| *I. punctatus* | 24 | 80,759 | 16,049,270 | 488 | 460 | 265 | | 28 | |  |
| *I. furcatus* | 24 | 5,861,679 | 20,110,004 | 444 | 424 | 268 | | 20 | |  |

**Supplementary Table S8. Numbers of genes on each of the 29 chromosomes of channel catfish and blue catfish**

| **Chromosome** | **Catfish Assembly** | | |
| --- | --- | --- | --- |
|  | Billie_1.0 | Coco_2.0 | Coco_1.2 |
| chr01 | 1146 | 1,171 | 1,090 |
| chr02 | 1217 | 1,258 | 1,135 |
| chr03 | 1023 | 1,039 | 958 |
| chr04 | 831 | 841 | 780 |
| chr05 | 1026 | 1,065 | 974 |
| chr06 | 883 | 893 | 816 |
| chr07 | 829 | 1,166 | 1,035 |
| chr08 | 899 | 925 | 886 |
| chr09 | 949 | 973 | 868 |
| chr10 | 674 | 854 | 788 |
| chr11 | 706 | 722 | 652 |
| chr12 | 954 | 1,020 | 858 |
| chr13 | 900 | 947 | 844 |
| chr14 | 891 | 893 | 764 |
| chr15 | 915 | 942 | 844 |
| chr16 | 873 | 818 | 747 |
| chr17 | 856 | 831 | 813 |
| chr18 | 861 | 871 | 825 |
| chr19 | 689 | 721 | 549 |
| chr20 | 702 | 706 | 657 |
| chr21 | 628 | 649 | 624 |
| chr22 | 660 | 674 | 654 |
| chr23 | 611 | 644 | 569 |
| chr24 | 672 | 734 | 661 |
| chr25 | 659 | 683 | 625 |
| chr26 | 722 | 726 | 687 |
| chr27 | 536 | 567 | 484 |
| chr28 | 662 | 730 | 646 |
| chr29 | 470 | 495 | 444 |
| Assigned | 23,444 | 24,558 | 22,277 |
| Unassigned | 102 | 477 | 823 |
| **Total** | **23,546** | **25,035** | **23,100** |

**Supplementary Table S9. Comparison of annotated protein coding genes from selected teleost species.** Channel catfish and blue catfish are highlighted.

| Scientific Name | Gene counts | Assembly Accession | Genome size (Mb) | # of Chr | BUSCO% |
| --- | --- | --- | --- | --- | --- |
| *P. marinus* | 17,567 | GCF_010993605.1 | 1089 | 86 | C:57.5%[S:52.6%,D:4.9%] |
| *L. oculatus* | 18,771 | GCF_000242695.1 | 945 | 30 | C:94.7%[S:93.6%,D:1.1%] |
| *O. latipes* | 22,058 | GCF_002234675.1 | 734 | 25 | C:98.3%[S:97.7%,D:0.6%] |
| *T. rubripes* | 22,063 | GCF_901000725.2 | 384 | 23 | C:98.3%[S:94.6%,D:3.7%] |
| *G. aculeatus* | 22,585 | GCF_016920845.1 | 467 | - | C:98.5%[S:95.9%,D:2.6%] |
| *S. meridionalis* | 22,599 | GCF_014805685.1 | 742 | 30 | C:97.5%[S:96.0%,D:1.5%] |
| *P. hypophthalmus* | 22,751 | GCF_009078355.1 | 742 | 30 | C:98.6%[S:97.3%,D:1.3%] |
| *H. wyckioides* | 22,794 | GCA_019097595.1 | 789 | 29 | C:94.8%[S:93.3%,D:1.5%] |
| *G. morhua* | 23,472 | GCF_902167405.1 | 669 | 24 | C:96.8%[S:95.0%,D:1.8%] |
| *I. furcatus* | 23,546 | Billie1.0 | 825 | 29 | C:95.6%[S:94.2%,D:1.4%] |
| *P. fulvidraco* | 23,998 | GCF_022655615.1 | 711 | 26 | C:98.8%[S:97.1%,D:1.7%] |
| *A. melas* | 24,354 | GCA_012411365.1 | 868 | 29 | C:91.9%[S:90.6%,D:1.3%] |
| *I. punctatus* | 25,035 | Coco2.0 | 842 | 29 | C:96.6%[S:95.5%,D:1.1%] |
| *A. mexicanus* | 25,117 | GCF_000372685.2 | 1335 | 25 | C:98.7%[S:97.7%,D:1.0%] |
| *D. rerio* | 25,432 | ENSEMBLE | 1373 | 25 | C:95.1%[S:93.2%,D:1.9%] |
| *O. niloticus* | 29,537 | GCA_001858045.3 | 1005 | 23 | C:99.7%[S:99.1%,D:0.6%] |

**Supplementary Table S10. Gene annotation of 508 gene families that were specific to channel catfish and blue catfish compared with other 14 representative fish species.**

Data provided in an external Excel file “Additional File 2.xlsx”.

**Supplementary Table S11.Annotation of 1127 genes within 732 families that are specific to channel catfish**

Data provided in an external Excel file “Additional File 2.xlsx”.

**Supplementary Table S12. Annotation of 606 genes within 434 families that are specific to blue catfish.**

Data provided in an external Excel file “Additional File 2.xlsx”.

**Supplementary Table S13. Categories of repetitive elements in the blue catfish (*Ictalurus furcatus*) genome**

| **Repeat** | **Length** | **%** |
| --- | --- | --- |
| DNA/TcMar-Tc1 | 83,151,469 | 22.12 |
| Simple_repeat | 34,731,662 | 9.24 |
| Immunoglobulin related repetitive proteins | 2906644 | 0.77 |
| Other repetitive proteins | 26,500,385 | 7.05 |
| LINE/L2 | 16,590,799 | 4.41 |
| DNA/hAT-Ac | 13,579,799 | 3.61 |
| LTR/Ngaro | 13,124,279 | 3.49 |
| LTR/Gypsy | 11,484,890 | 3.06 |
| LTR/DIRS | 10,251,231 | 2.73 |
| Uncharacterized DNA transposon | 8,981,475 | 2.39 |
| Repetitive non-coding RNAs | 6,284,437 | 1.67 |
| Xba | 1,304,797 | 0.35 |
| DNA/PIF-Harbinger | 5,376,076 | 1.43 |
| DNA/CMC-EnSpm | 5,210,653 | 1.39 |
| DNA/hAT-Charlie | 5,105,754 | 1.36 |
| LINE/Rex-Babar | 4,407,440 | 1.17 |
| LTR/ERV1 | 3,710,306 | 0.99 |
| SINE/MIR | 3,401,360 | 0.90 |
| RC/Helitron | 3,073,215 | 0.82 |
| Low_complexity | 3,040,409 | 0.81 |
| LINE/L1 | 2,808,496 | 0.75 |
| DNA/IS3EU | 2,765,346 | 0.74 |
| DNA/Crypton-A | 2,754,702 | 0.73 |
| DNA/hAT | 2,718,314 | 0.72 |
| SINE/tRNA-V-RTE | 2,661,977 | 0.71 |
| DNA/Kolobok-T2 | 2,490,206 | 0.66 |
| DNA/hAT-Tip100 | 2,428,893 | 0.65 |
| DNA/Maverick | 2,278,838 | 0.61 |
| LINE/L1-Tx1 | 1,961,698 | 0.52 |
| DNA/TcMar-Tigger | 1,717,466 | 0.46 |
| DNA/Crypton | 1,569,921 | 0.42 |
| LINE/RTE-BovB | 1,548,841 | 0.41 |
| DNA/hAT-Blackjack | 1,430,088 | 0.38 |
| LINE/I | 1,313,355 | 0.35 |
| DNA/Merlin | 1,187,644 | 0.32 |
| Satellite | 1,166,020 | 0.31 |
| SINE/5S-Deu-L2 | 1,029,311 | 0.27 |
| tRNA | 857,044 | 0.23 |
| LINE/RTE-X | 747,973 | 0.20 |
| DNA/TcMar | 674,779 | 0.18 |
| DNA/PIF-ISL2EU | 486,174 | 0.13 |
| DNA/MULE-NOF | 337,315 | 0.09 |
| DNA/PiggyBac | 326,193 | 0.09 |
| DNA/hAT-hAT5 | 300,289 | 0.08 |
| ARTEFACT | 260,193 | 0.07 |
| DNA/P | 257,481 | 0.07 |
| DNA/TcMar-ISRm11 | 257,430 | 0.07 |
| LTR/Pao | 248,760 | 0.07 |
| LINE/R2-Hero | 188,206 | 0.05 |
| DNA/Sola-1 | 167,187 | 0.04 |
| DNA/Crypton-V | 163,471 | 0.04 |
| LINE/Penelope | 141,582 | 0.04 |
| DNA/hAT-hobo | 124,048 | 0.03 |
| DNA/Sola-2 | 120,239 | 0.03 |
| SINE | 114,314 | 0.03 |
| DNA/TcMar-Tc2 | 101,942 | 0.03 |
| DNA/PIF | 77,674 | 0.02 |
| DNA/MULE-MuDR | 71,070 | 0.02 |
| DNA/CMC-Chapaev-3 | 51,023 | 0.01 |
| snRNA | 43,962 | 0.01 |
| LINE/Dong-R4 | 33,486 | 0.01 |
| DNA/TcMar-Mariner | 28,317 | 0.01 |
| Retroposon | 14,819 | 0.00 |
| DNA/hAT-hAT6 | 7,579 | 0.00 |
| SINE? | 3,818 | 0.00 |
| LINE | 39 | 0.00 |
| Unknown | 73,611,553 | 19.58 |

**Supplementary Table S14. Categories of repetitive elements in the channel catfish (*Ictalurus punctatus*) genome**

| **Repeat** | **Length** | **%** |
| --- | --- | --- |
| DNA/TcMar-Tc1 | 82,901,706 | 20.64 |
| Simple_repeat | 42,717,594 | 10.64 |
| Immunoglobulin related repetitive proteins | 4,828,095 | 1.20 |
| Other repetitive proteins | 27,007,585 | 6.73 |
| LINE/L2 | 17,808,121 | 4.43 |
| LTR/Ngaro | 16,315,882 | 4.06 |
| DNA/hAT-Ac | 13,984,821 | 3.48 |
| LTR/Gypsy | 12,898,396 | 3.21 |
| LTR/DIRS | 9,648,552 | 2.40 |
| Uncharacterized DNA transposon | 9,574,292 | 2.38 |
| Repetitive non-coding RNAs | 8,393,149 | 2.09 |
| Xba | 6,735,401 | 1.68 |
| DNA/CMC-EnSpm | 5,739,945 | 1.43 |
| DNA/hAT-Charlie | 5,558,873 | 1.38 |
| LINE/Rex-Babar | 4,652,774 | 1.16 |
| DNA/PIF-Harbinger | 4,550,167 | 1.13 |
| DNA/Crypton-A | 4,172,845 | 1.04 |
| LTR/ERV1 | 3,896,973 | 0.97 |
| LINE/L1-Tx1 | 3,531,873 | 0.88 |
| SINE/MIR | 3,431,555 | 0.85 |
| RC/Helitron | 3,100,451 | 0.77 |
| Low_complexity | 2,913,787 | 0.73 |
| DNA/IS3EU | 2,787,048 | 0.69 |
| LINE/L1 | 2,739,583 | 0.68 |
| DNA/hAT | 2,664,618 | 0.66 |
| tRNA | 2,367,818 | 0.59 |
| DNA/Maverick | 2,302,299 | 0.57 |
| DNA/Kolobok-T2 | 2,048,417 | 0.51 |
| DNA/hAT-Tip100 | 1,767,896 | 0.44 |
| DNA/TcMar | 1,546,743 | 0.39 |
| Satellite | 1,528,735 | 0.38 |
| DNA/hAT-Blackjack | 1,397,768 | 0.35 |
| LINE/RTE-BovB | 1,363,768 | 0.34 |
| LINE/Penelope | 1,107,605 | 0.28 |
| SINE/5S-Deu-L2 | 1,010,122 | 0.25 |
| DNA/TcMar-Tigger | 854,673 | 0.21 |
| LINE/I | 793,650 | 0.20 |
| SINE/tRNA-V-RTE | 662,794 | 0.17 |
| SINE? | 651,403 | 0.16 |
| DNA/Merlin | 497,880 | 0.12 |
| DNA/PIF-ISL2EU | 482,858 | 0.12 |
| DNA/hAT-hAT5 | 430,237 | 0.11 |
| LTR/ERVK | 420,050 | 0.10 |
| DNA/PiggyBac | 407,900 | 0.10 |
| LINE/RTE-X | 392,159 | 0.10 |
| LTR | 359,211 | 0.09 |
| DNA/TcMar-Tc2 | 292,490 | 0.07 |
| rRNA | 281,701 | 0.07 |
| LINE/R2-Hero | 277,415 | 0.07 |
| DNA/MULE-NOF | 267,700 | 0.07 |
| DNA/TcMar-Mariner | 251,897 | 0.06 |
| SINE/B2 | 244,496 | 0.06 |
| DNA/P | 212,411 | 0.05 |
| LTR/Pao | 199,092 | 0.05 |
| DNA/hAT-hobo | 168,437 | 0.04 |
| DNA/TcMar-ISRm11 | 165,836 | 0.04 |
| DNA/Zisupton | 138,465 | 0.03 |
| DNA/Sola-1 | 126,870 | 0.03 |
| DNA/PIF | 72,612 | 0.02 |
| SINE | 70,279 | 0.02 |
| DNA/hAT-hATx | 55,336 | 0.01 |
| DNA/CMC-Chapaev-3 | 48,112 | 0.01 |
| DNA/Sola-2 | 44,224 | 0.01 |
| LINE/R2 | 35,704 | 0.01 |
| DNA/Crypton | 25,818 | 0.01 |
| SINE/Alu | 18,026 | 0.00 |
| DNA/MULE-MuDR | 16,882 | 0.00 |
| snRNA | 6,756 | 0.00 |
| Retroposon | 4,520 | 0.00 |
| DNA/Kolobok | 271 | 0.00 |
| Unknown | 73,600,694 | 18.33 |

**Supplementary Table S15. Centromere positions in Blue and Channel Catfish chromosomes measured as span of Xba elements**.

|  | **Blue catfish** | | **Channel catfish** | |  |
| --- | --- | --- | --- | --- | --- |
| **Chromosome** | **Begin** | **End** | **Begin** | **End** |  |
| Chr01 | 5,802,852 | 5,819,039 | 5,507,393 | 5,587,296 |  |
| Chr02 | 8,325,490 | 8,531,188 | 8,058,617 | 8,528,770 |  |
| Chr03 | 10,225,842 | 10,253,906 | 10,303,629 | 10,350,371 |  |
| Chr04 | 17,214,596 | 17,408,687 | 16,984,395 | 17,158,113 |  |
| Chr05 | 14,371,019 | 14,402,295 | 14,524,817 | 15,604,449 |  |
| Chr06 | 5,629,335 | 5,659,026 | 8,797,570 | 8,903,713 |  |
| Chr07 | 5,968,321 | 6,006,367 | 4,398,737 | 4,530,591 |  |
| Chr08 | 6,811,355 | 6,999,707 | 6,628,228 | 6,710,732 |  |
| Chr09 | 6,676,492 | 6,709,564 | 6,848,984 | 7,383,691 |  |
| Chr10 | 3,032,007 | 3,058,195 | 1,924,785 | 2,349,867 |  |
| Chr11 | 13,389,729 | 13,579,576 | 1,869,783 | 1,967,594 |  |
| Chr12 | 2,910,090 | 2,934,366 | 6,453,982 | 6,727,061 |  |
| Chr13 | 6,673,823 | 6,688,538 | 6,570,667 | 6,967,611 |  |
| Chr14 | 4,603,146 | 4,631,624 | 2,747,407 | 2,909,316 |  |
| Chr15 | - | - | 103 | 27,132 |  |
| Chr16 | 9,315,744 | 9,337,531 | 9,160,411 | 9,395,931 |  |
| Chr17 | 154 | 13,532 | 27,946 | 49,909 |  |
| Chr18 | 9,748,354 | 9,772,297 | 9,685,601 | 9,806,533 |  |
| Chr19 | 3,707,454 | 3,734,994 | 4,539,959 | 4,656,040 |  |
| Chr20 | 9,466,636 | 9,498,889 | 9,450,376 | 10,470,460 |  |
| Chr21 | 1 | 19,901 | 1 | 32,020 |  |
| Chr22 | 5,115,524 | 5,336,084 | 4,910,846 | 5,083,579 |  |
| Chr23 | 10,255,956 | 10,505,525 | 10,006,289 | 10,105,978 |  |
| Chr24 | 7,732,734 | 7,744,143 | 3,330,542 | 3,732,755 |  |
| Chr25 | 5,120,549 | 5,148,735 | 4,719,529 | 4,789,784 |  |
| Chr26 | 6,458,946 | 6,708,270 | 6,975,619 | 7,087,928 |  |
| Chr27 | 5,362,420 | 5,378,264 | 5,330,427 | 5,459,582 |  |
| Chr28 | 8,212,303 | 13,329,206 | 8,160,828 | 8,251,713 |  |
| Chr29 | 6,729,103 | 6,757,779 | 6,459,290 | 6,542,864 |  |

**Supplementary Table S16. Centromeric and telomeric sequences for channel and blue catfish chromosome assemblies.** Position numbers in red are highlighted as different between channel and blue.

| **Chrom** | **Coco_2.0** | | | | |  | | **Billie_1.0** | | | | | |  | |  |
| --- | --- | --- | --- | --- | --- | --- | --- | --- | --- | --- | --- | --- | --- | --- | --- | --- |
|  | Centromere | | Telomere | | | |  | | Centromere | | Telomere | | | |  | |
|  | Length^1^ | 5’ | | 3’ | Pos^2^ | Ori | | Length | | 5’ | | 3’ | Pos^2^ | Ori | |  |
| Chr01 | (79,903) | • | | • | 14.1 | F | | (16,187) | | • | | • | 14.6 | F | |  |
| Chr02 | 470,153 |  | | • | 20.0 | R | | 205,698 | | • | | • | 20.7 | R | |  |
| Chr03 | (46,672) |  | | • | 27.3 | F | | (28,064) | | • | | • | 26.7 | F | |  |
| Chr04 | (173,718) | • | | • | 47.1 | F | | 194,091 | | • | | • | 46.3 | F | |  |
| Chr05 | 1,079,632 | • | | • | 41.1 | F | | (31,276) | |  | | • | 41.9 | F | |  |
| Chr06 | (106,143) | • | | • | 25.9 | R | | (29,691) | | • | | • | 15.6 | R | |  |
| Chr07 | (131,854) | • | | • | 12.6 | R | | (38,046) | | • | | • | 16.7 | R | |  |
| Chr08 | (82,504) | • | | • | 21.1 | F | | 188,352 | | • | | • | 21.6 | F | |  |
| Chr09 | 534,707 | • | | • | 21.1 | F | | (33,072) | | • | | • | 20.9 | F | |  |
| Chr10 | 425,082 |  | | • | 6.3 | R | | (26,188) | |  | | • | 9.4 | R | |  |
| Chr11 | (97,811) |  | | • | 6.6 | R | | 189,847 | | • | | • | 46.7 | R | |  |
| Chr12 | 273,079 | • | | • | 19.1 | R | | (24,276) | |  | | • | 9.3 | R | |  |
| Chr13 | 396,944 | • | | • | 22.0 | R | | (14,715) | |  | | • | 22.1 | R | |  |
| Chr14 | (161,839) | • | | • | 9.4 | R | | (28,478) | | • | | • | 14.4 | R | |  |
| Chr15 | 27,029^3^ |  | | • | 0.00 | R | | - | |  | | • | 0.00 |  | |  |
| Chr16 | (235,520) | • | | • | 33.8 | R | | (21,787) | | • | | • | 33.6 | R | |  |
| Chr17 | 21,963^3^ |  | | • | 0.1 | F | | (13,378) | |  | | • | 0.00 | F | |  |
| Chr18 | (120,932) | • | | • | 38.5 | F | | (23,943)^2^ | | • | | • | 38.2 | F | |  |
| Chr19 | (116,081) | • | | • | 16.2 | F | | (27,610) | | • | | • | 13.2 | F | |  |
| Chr20 | 1,020,084 | • | | • | 37.8 | R | | (31,699) | | • | | • | 39.1 | R | |  |
| Chr21 | 32,018^2^ |  | | • | 0.00 | R | | (19,566) | |  | | • | 0.00 | R | |  |
| Chr22 | (172,733) | • | | • | 23.7 | F | | 220,071 | | • | |  | 24.3 | R | |  |
| Chr23 | (99,689) | • | | • | 48.1 | R | | 249,569 | | • | |  | 48.0 | R | |  |
| Chr24 | 402,213 | • | | • | 15.0 | F | | (11,409) | | • | | • | 37.9 | F | |  |
| Chr25 | (70,265) | • | | • | 23.3 | F | | (28,186) | | • | | • | 24.1 | F | |  |
| Chr26 | (112,309) | • | | • | 33.7 | R | | 249,394 | | • | | • | 30.9 | F | |  |
| Chr27 | 129,155 | • | | • | 27.4 | R/F | | (15,351) | | • | | • | 26.8 | R | |  |
| Chr28 | (95,052) | • | | • | 40.6 | F | | (18,722) | | • | |  | 41.9 | F | |  |
| Chr29 | (83,574) | • | | • | 39.2 | F | | (28,676) | | • | | • | 39.6 | F | |  |
| Avg Length | **481,277** |  | |  |  |  | | **219,446** | |  | |  |  |  | |  |

^1^ Length in parentheses = chromosome arms scaffolded at centromere with 100 bp gap

^2^ Pos = centromere position as percentage of chromosome length

^3^ Centromere at 5’ end of chromosome assembly

**Supplementary Table S17. Primers and expected amplicons of junction PCR.**

|  | **Breakpoint** | **Forward primer** | **Reverse primer** | **Expected Amplicon (bp)** | |
| --- | --- | --- | --- | --- | --- |
|  | **position** |  |  | Blue | Channel |
| **Chromosome 6** | | | | | |
| Blue (BEG) | 468,710 | P1:TGTGCGTAACGCTAACGAGAAGA | P2:CCCCAACAGCTAATTTTCCCTACA | 814 | - |
| Channel (BEG) | 3,815,287 | P5:CATTGCACTCACTGACAAGAACAT | P2:CCCCAACAGCTAATTTTCCCTACA | - | 920 |
| Blue (END) | 30,277,155 | P3:TAGTTGTGGTCCACTGATGTGATT | P6:GCGGGTATACTGAATATCGGAAGA | 490 | - |
| Channel (END) | 33,476,410 | P3:TAGTTGTGGTCCACTGATGTGATT | P4:TTGAAGTGTCAAAGGGAGGGTTAG | - | 752 |
| **Chromosome 11** | | | | | |
| Blue (BEG) | 11,638,545 | P5:TCACAAGCTCATCGGTTCCT | P2:TAGTGTTCCGCTGGCTTCAT | 480 | - |
| Channel (BEG) | 218,068 | P1:TACGAGCCCAGTGTCGTAGA | P2:TAGTGTTCCGCTGGCTTCAT | - | 271 |
| Blue (END) | 28,638,249 | P3:AACAAGTTAATGGCCTTGTGAGTG | P6:GTAAAACTACCCCAATACAACCGC | 496 | - |
| Channel (END) | 16,917,745 | P3:AACAAGTTAATGGCCTTGTGAGTG | P4:AAAGCAACCTGATTTTCATTGGGT | - | 464 |
| **Chromosome 24** | | | | | |
| Blue (BEG) | 5,861,680 | P5:ACTGTTTCTCCGCTGTATCAATCA | P2:GAAATGCGTGTGCTTCTGGCAA | 691 | - |
| Channel (BEG) | 80,759 | P1:CGTAAAAGAACGGAAGCCCCATT | P2:GAAATGCGTGTGCTTCTGGCAA | - | 997 |
| Blue (END)* | 20,110,005 | - | - | - | - |
| Channel (END)* | 16,049,270 | - | - | - | - |

* Primers could not be designed for the end of chromosome 24 due to repetitive DNA.
